# Regional economic integration via detection of circular flow in international value-added network

**DOI:** 10.1371/journal.pone.0255698

**Published:** 2021-08-20

**Authors:** Sotaro Sada, Yuichi Ikeda

**Affiliations:** Graduate School of Advanced Integrated Studies in Human Survivability, Kyoto University, Kyoto, Japan; Unviersity of Burgundy, FRANCE

## Abstract

Global value chains are formed through value-added trade, and some regions promote economic integration by concluding regional trade agreements to promote these chains. However, it has not been established to quantitatively assess the scope and extent of economic integration involving various sectors in multiple countries. In this study, we used the World Input–Output Database to create a cross-border sector-wise network of trade in value-added (international value-added network) covering the period of 2000–2014 and evaluated them using network science methods. By applying Infomap to the international value-added network, we confirmed two regional communities: Europe and the Pacific Rim. We applied Helmholtz–Hodge decomposition to the value-added flows within the region into potential and circular flows, and clarified the annual evolution of the potential and circular relationships between countries and sectors. The circular flow component of the decomposition was used to define an economic integration index. Findings confirmed that the degree of economic integration in Europe declined sharply after the economic crisis in 2009 to a level lower than that in the Pacific Rim. The European economic integration index recovered in 2011 but again fell below that of the Pacific Rim in 2013. Moreover, sectoral economic integration indices suggest what Europe depends on Russia in natural resources makes the European economic integration index unstable. On the other hand, the indices of the Pacific Rim suggest the steady economic integration index of the Pacific Rim captures the stable global value chains from natural resources to construction and manufactures of motor vehicles and high-tech products.

## Introduction

It is not easy to grow a country’s economy without establishing economic relations with other countries. Every year, the amount of international trade increases along with the world’s GDP. Free trade agreements (FTAs) and regional trade agreements (RTAs) have been created to support this trend, and countries are working to stabilize trade.

How, then, do countries become interdependent through trade? Classically, Ricard advocated comparative advantage, which stated that countries specializing in different industries are supposed to trade by taking advantage of their respective strengths [1]. However, in recent years, trade in intermediate goods, which did not exist at that time, has begun and flourished. In other words, there are forms of trade specializing in different industries and global value chains (GVCs) in the same industry to produce complex and sophisticated products. In addition, distance (as represented by the gravity model) and economic integration (such as that promoted by the EU and NAFTA) facilitate international trade.

Many studies have been conducted on measuring international production structures. In the 21st century, the measurement of GVCs became a widely discussed topic after Hummels et al. [2] proposed the vertical specialization index. Moreover, the VAX and FVAiX indices were proposed by Johnson and Noguera [3], and Amador et al. [4], respectively. Koopman et al. [5] decomposed and classified the trade prices into nine terms and clarified double-counted terms.

The abovementioned progress has led to the development of international input–output (IO) tables. After Dietzenbacher et al. [6] and Timmer et al. [7, 8] publicized the World Input–Output Database (WIOD), Cerina et al. [9] and Zhu et al. [10] analyzed the data for measuring GVCs network analysis methods. Los et al. [11] showed that regional value chains had been fragmented more rapidly than global ones in 1995–2011. According to Johnson and Noguera [12], over 40 years, the ratio of value-added to gross exports in non-manufacturing sectors rose, while that in manufacturing sectors fell by 20 percentage points; their study showed that RTAs led to such decline.

Due to international relationship problems in the economy, researchers in international organizations have been analyzing GVCs. Recently comprehensive summary reports of GVCs research have been published by the World Bank [13, 14]. According to these reports, GVCs research aims to address the misunderstanding of trade data and give international trade opportunities and export diversity to developing countries for economic growth [15]. As shown by Degain et al., GVCs had been expanding until the global financial crisis, but they stopped growing in 2011 when they recovered from a decline in equipment. The GVCs expansion seen before the financial crisis has been interpreted as China’s accession to the WTO and participation in GVCs [15]. These GVCs research developments were described in detail by Amador and Cabral [16], and Inomata [17]. However, economics studies mainly focus on the relationship between nations. Little works are showing the complicated relationship between industries in different countries.

By contrast, there are many studies on international trade in network science because of recent developments in analytical methods and data availability. Initially, the scale-free characteristics of international trade networks were clarified [18–20]; then, the virtual nodes in these networks were examined using centrality indices and other measures, and changes over time were analyzed [21–23].

Ikeda et al. [24, 25] studied the international trade network of the WIOD using community analysis of modularity maximization, which combines the synchronization aspect of the G7 production network [26, 27]; then, they found stable sectoral communities that had emerged in 1995–2011.

From an institutional point of view, a study examining interregional agreements and geographic factors in international trade change found no evidence that the WTO has contributed to the increase in international trade and indicated that geographic factors are more significant than interregional agreements [28]. By contrast, other studies show that trade policies contribute to shaping trade networks [29], and the impact of distance by trade is decreasing in industrial sectors [30].

However, there is no way to quantitatively assess the scope and extent of economic integration involving various sectors in multiple countries. It is not easy to quantitatively evaluate such economic phenomena, where multiple countries and sectors are intertwined, and the trade balance between two countries is still used as a basis for foreign economic policy. In this study, we analyze the economic linkages through trade that have been made in recent years using international IO tables and network science methods and clarify how the world economy is being integrated from the perspective of value-added trade.

In this context, this study offers the following essential contributions. First, rather than using trade figures, which can mislead international economic relations, we used the value-added calculations used in an IO analysis to construct a sector-wise network. Next, from the value-added linkage, we confirmed the regional characteristics of Europe and the Pacific Rim. Findings showed that the behavior of this value-added relationship is highly different from that of the sectoral community in the international trade network shown in previous studies. The dense economic relationships within these regions were decomposed into potential and circular relationships, and the countries and sectors that contribute to the strong regional ties were identified. Finally, we propose an economic integration index based on the circular flow component, and the results showed that economic integration was higher in the Pacific Rim than in Europe in 2010, 2013, and 2014.

The remainder of this paper is organized into three parts. The following section describes the IO table and the computational method used to create the network and the community analysis, explains the decomposition of the potential and circular flow components, and presents the proposed method for measuring economic integration. Then, the characteristics and structure of the network, the potential and circular relationships, and the results of the integration index are presented, and the international economic linkages are examined based on these results. Finally, we conclude this study.

## Data and methods

This section consists of four subsections. First, we describe the WIOD and the computational methods used to build the cross-border, sector-wise trade in value-added network (international value-added network, IVAN). Second, we briefly describe Infomap, a community analysis algorithm, which was used to determine the extent to which economic integration is occurring. Third, we explain the use of Helmholtz–Hodge decomposition to extract the potential and circular nature of IVANs. Finally, the economic integration index is defined.

### Data

We used the WIOD released in 2016, which includes 43 countries, and classifies the rest of the world (RoW) into 56 sectors. These countries and sectors are listed in S1 and S2 Tables. The country codes are the same as those of the WIOD, but we simplified the sectoral codes. The WIOD has IO tables for 2000–2014. Table 1 shows a simplified example of the table. The IO table was developed by Leontief [31] and is now used globally to estimate economic and environmental conditions. In this paper, we used the WIOD without RoW because we aim to analyze the relationships of specific countries.

**Table 1:** World input–output table with two sectors in three countries.

		Intermediate demand						Final demand			Total output
		Country A		Country B		Country C					
		Sector 1	Sector 2	Sector 1	Sector 2	Sector 1	Sector 2	Country A	Country B	Country C	
Country A	Sector 1	*Z*_1,1_	*Z*_1,2_	*Z*_1,3_	*Z*_1,4_	*Z*_1,5_	*Z*_1,6_	*F*_1,1_	*F*_1,2_	*F*_1,3_	*T*_1_
	Sector 2	*Z*_2,1_	*Z*_2,2_	*Z*_2,3_	*Z*_2,4_	*Z*_2,5_	*Z*_2,6_	*F*_2,1_	*F*_2,2_	*F*_2,3_	*T*_2_
Country B	Sector 1	*Z*_3,1_	*Z*_3,2_	*Z*_3,3_	*Z*_3,4_	*Z*_3,5_	*Z*_3,6_	*F*_3,1_	*F*_3,2_	*F*_3,3_	*T*_3_
	Sector 2	*Z*_4,1_	*Z*_4,2_	*Z*_4,3_	*Z*_4,4_	*Z*_4,5_	*Z*_4,6_	*F*_4,1_	*F*_4,2_	*F*_4,3_	*T*_4_
Country C	Sector 1	*Z*_5,1_	*Z*_5,2_	*Z*_5,3_	*Z*_5,4_	*Z*_5,5_	*Z*_5,6_	*F*_5,1_	*F*_5,2_	*F*_5,3_	*T*_5_
	Sector 2	*Z*_6,1_	*Z*_6,2_	*Z*_6,3_	*Z*_6,4_	*Z*_6,5_	*Z*_6,6_	*F*_6,1_	*F*_6,2_	*F*_6,3_	*T*_6_
Value-added		*V*_1_	*V*_2_	*V*_3_	*V*_4_	*V*_5_	*V*_6_				
Total output		*T*_1_	*T*_2_	*T*_3_	*T*_4_	*T*_5_	*T*_6_				

### Calculation for adjacency matrix of IVAN

Let *n*_*c*_ be the number of countries and *n*_*s*_ be the number of sectors in the IO table. Then, we use the *n*_*c*_ *n*_*s*_ × *n*_*c*_ *n*_*s*_ intermediate matrix **Z**, *n*_*c*_ *n*_*s*_ × *n*_*c*_ final demand matrix **F**, 1 × *n*_*c*_ *n*_*s*_ value-added vector **V**, and 1 × *n*_*c*_ *n*_*s*_ total output matrix **T** from WIOD. We calculate the value-added coefficient vector **C**, the final demand vector **D**, and the coefficient matrix **A** as **V**/(**T**)^⊤^, *D*_*j*_ = ∑_*i*_ *F*_*ij*_, and **Z**/**T**^⊤^, respectively. **T**^⊤^ means transpose of the vector **T**. Then, we calculate an induced value-added vector $\hat{C}LD$ by a vector **LD** left-hand multiplied by the *n*_*c*_ *n*_*s*_ × *n*_*c*_ *n*_*s*_ diagonal matrix $\hat{C}$, where the diagonal components are the vector **C** and **L** is well-known Leontief matrix which calculated by (**I** − **A**)^−1^, where **I** is the identity matrix. In this paper, we use ˆ as a symbol of diagonal matrix.

Then, the adjacency matrix of the global value-added network (GVAN) **G** is calculated as $\hat{C}L\hat{D}$, where $\hat{D}$ is a diagonal matrix whose diagonal components are the vector **D**. This operation $\hat{C}L\hat{D}$ calculates the value called trade in value-added (TiVA) [32]. Eliminating components of 43 on-diagonal 56 × 56 blocks (which mean domestic components) as zero from **G**, we obtained the IVAN’s adjacency matrix **Y**. This matrix represents the sum of all the ripple effects of value-added induced by the final demand in foreign sectors. The GVAN and IVAN nodes comprise 56 sectors in 43 countries. S1 and S2 Tables list these countries and sectors.

In summary, IVANs are directed and weighted networks constructed by the adjacency matrix **Y**, which does not have domestic links. By contrast, GVANs have domestic and international links. In the example of Table 1, **Y** is written as

$$\begin{array}{cc} Y & =(\begin{array}{cccccc} 0 & 0 & G_{1,3} & G_{1,4} & G_{1,5} & G_{1,6}\\ 0 & 0 & G_{2,3} & G_{2,4} & G_{2,5} & G_{2,6}\\ G_{3,1} & G_{3,2} & 0 & 0 & G_{3,5} & G_{3,6}\\ G_{4,1} & G_{4,2} & 0 & 0 & G_{3,5} & G_{3,6}\\ G_{5,1} & G_{5,2} & G_{5,3} & G_{5,4} & 0 & 0\\ G_{6,1} & G_{6,2} & G_{6,3} & G_{6,4} & 0 & 0 \end{array}). \end{array}$$ (1)

### Community detection

To know how each network has developed a community structure, we apply community analysis to IVANs. Some community analysis methods have been developed, and their features were summarized by Fortunato and Hric [33] and Barabási [34]. We used Infomap, which is an application of random walk and Huffman coding [35] to analyze the communities in the studied network [36]. Let a random walker run in the network where this analysis will be applied; the random walker transitions with a probability dependent on the path weights (plus a constant transposition probability) [37]. The map equation establishes communities and renames the node in which the random walk track’s code length is minimized. In comparison, the well-known method of modularity maximization clusters a network by counting nodes’ link weights, inflows, and outflows, whereas the map equation method clusters a network by the remaining time of the random walker in the nodes; this difference leads to variations in results [36, 38].

In the Infomap method, the optimization problem for community segmentation in a network is replaced with the minimization problem of the code length of a segmented network. Consider the segmentation of a network composed of *n* nodes into *m* communities with a community partition M. Let the mean code length of segmented communities (index code length) be H(Q) and the mean code length of nodes within a community *i* be $H(P^{i})$; then, with use of Shannon’s source coding theorem [39], the average description length of a single step of the random walk *L*(*M*) is calculated as follows:

$$\begin{array}{c} L\left(M\right)=q_{↷}H\left(Q\right)+\sum_{i=1}^{m}p_{↻}^{i}H\left(P^{i}\right), \end{array}$$ (2)

where *q*_↷_ is the probability of the random walker moving to another community and $p_{↻}^{i}$ is the probability of the random walker moving within a community *i* = 1, 2, …, *m* plus the exit probability from *i*. Each of the probabilities is defined as $q_{↷}=\sum_{i=1}^{m}q_{i↷}$ and $p_{↻}^{i}=\sum_{\alpha \in i}p_{\alpha }+q_{i↷}$, where *p*_*α*_ is the probability of visiting node *α* = 1, 2, …, *n* and *q*_*i*↷_ is the exit probability from community *i*. Eq (2) is called the map equation [38].

### Structural characteristics of networks

Specific indices reveal network characteristics. We used eleven indices: (1) density, reciprocity, diameter, average path length, average betweenness, assortativity, average in-degree, and average out-degree are calculated as unweighted and directed networks; (2) in-strength and out-strength are for weighted and directed ones; (3) the clustering coefficient is calculated as unweighted and undirected [34].

The density of a network is the proportion of the number of links to the number of maximum possible links in the network. Thus, it is calculated as *l*/*n*(*n* − 1), where *n* and *l* are the numbers of nodes and links in the network, respectively.

The reciprocity of a network is the proportion of the reciprocal links to the total number of links in the network. It is calculated as *l*_*m*_/*l*, where *l*_*m*_ is the number of reciprocal links (two directed links between two nodes in the network).

The clustering coefficient indicates how each node is connected to its neighbors. The clustering coefficient *c*_*i*_ of a node *i* is calculated as *c*_*i*_ = *l*_*i*_/*k*_*i*_(*k*_*i*_ − 1), where *l*_*i*_ (*k*_*i*_) is the number of links (neighbors) of node *i*. Thus, the clustering coefficient of a network is ∑*c*_*i*_/*n*.

The diameter of a network is the maximum number of shortest paths for all pairs of nodes in the network.

The average path length of a network is the mean of the shortest path length in the network: ∑_*i*,*j*;*i*≠*j*_ *d*_*ij*_/*n*(*n* − 1), where *d*_*ij*_ is the shortest path length between nodes *i* and *j*.

The betweenness is calculated by the number of the shortest paths through a node. The betweenness *b*_*i*_ of node *i* is ∑_*j* ≠ *k* ≠ *i*_ *s*_*jk*_(*i*)/*s*_*jk*_, where *s*_*jk*_ is the number of the shortest paths between nodes *j* and *k*, and *s*_*jk*_(*i*) is the number of the shortest paths through node *i*. Therefore, the average betweenness of a network is the mean of *b*_*i*_.

The degree of a node means the sum of the number of links in the node. There are two kinds of degrees in a directed network, namely, in-degree and out-degree, which are the sums of the links to the node from the other nodes (inflow) and links from the node to other nodes (outflow), respectively. Thus, the in-degree and out-degree are represented as: ∑_*j*_ *l*_*j*,*i*_ and ∑_*j*_ *l*_*i*,*j*_, respectively, where *l*_*i*,*j*_ is a link (flow) from node *i* to node *j*.

The assortativity of a network scores the similarity of the connections in the network as -1 to 1, which is calculated by (∑_*jk*_ *jke*_*jk*_ − *μ*)/*σ*^2^, where *e*_*jk*_ is the probability of the two nodes of degrees *j* and *k* are at the end of a randomly chosen link, and *μ* and *σ* are the mean and standard deviation, respectively, of the excess degree distribution. Assortativity close to -1 (1) indicates that high-degree nodes in a network are connected to low-degree nodes (high-degree nodes).

The strength of a node is the total weight of the links in the node. There are two kinds of strength in a directed network, namely, in-strength and out-strength, which are the summed weights of the inflow and outflow, respectively. Thus, the in-strength and out-strength are represented as: $\sum_{j}l_{j,i}^{w}$ and $\sum_{j}l_{i,j}^{w}$, respectively, where $l_{i,j}^{w}$ is the flow amount from node *i* to node *j*.

### Decomposition to potential and circular flows

Firstly, we define flows as directed and weighted links, and the flow of IVANs as value flow. We used Helmholtz–Hodge decomposition [40] to extract potential and circular relationships from IVANs. This method was also used by Kichikawa et al. [41] to clarify the potential relationships in corporate transaction networks. With Helmholtz–Hodge decomposition, the flow *F*_*ij*_ from node *i* to node *j* can be separated into the circular flow $F_{ij}^{\left(c\right)}$ and potential flow $F_{ij}^{\left(p\right)}:F_{ij}=F_{ij}^{\left(c\right)}+F_{ij}^{\left(g\right)}$. Here the potential flow $F_{ij}^{\left(p\right)}$ is given by $F_{ij}^{\left(p\right)}=w_{ij}\left(\phi_{i}-\phi_{j}\right)$, where *ϕ*_*i*_ is the Helmholtz–Hodge potential and *w*_*ij*_ is a positive weight for the link between node *i* and node *j*. The circular flow satisfies $\sum_{j}F_{ij}^{\left(c\right)}=0$, in which the inflows and outflows are balanced in each node. $F_{ij}^{\left(p\right)}$ represents the difference in potentials between the nodes, whereas $F_{ij}^{\left(c\right)}$ represents the number of feedback loops among the nodes.

An IVAN captures the value-added chain of various international sectors to their respective final goods. The potential of a sector is the difference between the amount of value added by the sector for final foreign production and the amount of value added by the foreign sector for the sector’s final production. In other words, if the value-added potential is positive, the sector contributes more to the production of the final goods of the foreign sector, and if it is negative, the sector is contributed more to the own production of the final goods by foreign sectors. This can indicate the degree of asymmetric dependence or co-dependence. Moreover, the circular flow component indicates the amount of contribution to (from) the foreign sector, that is, the degree of interdependence.

### Economic integration index

When several economies proceed with economic integration by some factors such as establishing FTAs or RTAs, the value added in a country becomes increasingly induced by other countries, and vice versa. In other words, higher economic integration makes a larger feedback loop within an IVAN. Based on this assumption, we define the economic integration index *E* as the aggregate amount of circular flow divided by the total flow of the GVAN in a specific community.

$$\begin{array}{c} E=\frac{\sum_{i>j}Y_{ij}^{\left(c\right)}}{\sum G_{ij}} \end{array}$$ (3)

The range of applications of this index covers communities detected by Infomap, which also detects communities using flow. The detected communities are interpreted as the circulation observed when random walkers rotate in the community. The degree of circulation is quantified and divided by the economic scale of the community; then, the economic integration is measured as the amount of value-added circulation per economic scale.

The index defined here indicates how much of the domestic and international value-added chain toward final demand can be extracted as international interdependence. The higher the value of the index, the greater the value-added induced in the community across national borders (economic activities performed by sectors in other countries), and this value measures economic integration in this study.

This economic integration index can decompose into sectoral indices arbitrarily. For sector *k*, the sectoral economic integration index is

$$\begin{array}{c} E_{k}=\frac{\sum_{i>j;i,j\in S_{k}}Y_{ij}^{\left(c\right)}}{\sum_{i,j\in S_{k}}G_{ij}}, \end{array}$$ (4)

where *S*_*k*_ is a set of nodes in sector *k*.

In summary, the economic integration index is the circular magnitude of the cross-border value-added contribution of economic activities in a country (other countries) toward the final production of sectors in other countries (home country), compared with the circular magnitude of the value-added contribution of economic activities in the community, including circulation within a country.

## Results

This section is composed of three subsections. The first subsection shows the community structure of the IVANs, the threshold set for community detection, and the resulting 15-year change in regional communities. The second subsection shows the essential characteristics of the original IVAN, the cut IVAN, and the IVAN of the regional communities in selected years. The third subsection confirms the value-added potential and circular relationships revealed by Helmholtz–Hodge decomposition for countries and sectors in Europe and the Pacific Rim. The last subsection shows the evolution of economic integration in the regional communities and the results of decomposing the economic integration indicators into key sectoral components.

### Communities of IVANs

**Fig 1:**
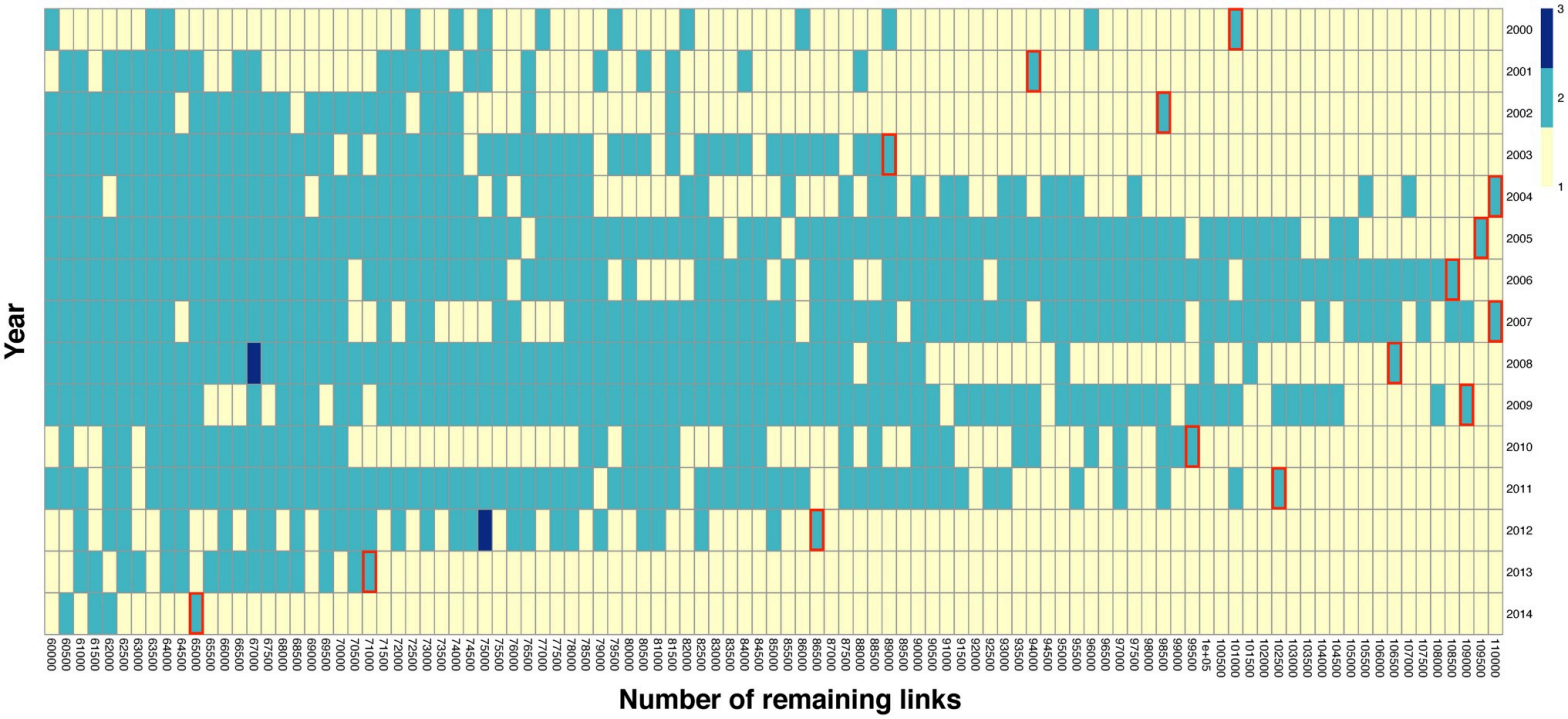
Heat map of the number of major communities in the IVANs cut by each threshold, in 2000–2014. This research uses the thresholds represented as a red-bordered cell in each year. Colors mean the number of major communities.

Infomap detected only a giant community from IVAN without thresholds for 15 years. This result differs from the community of international trade networks (see S3 Table and S1 Fig) also shown in a previous study [24] which illustrated 7–12 communities. Next, thresholds are set to exclude the branches and leaves of the IVANs to see any concealed community structure. In the range of 6,500–11,000 remaining links, several large communities appear (Fig 1). Here, the thresholds are set by the number of the most extensive links to be retained, as the threshold value set by USD is inappropriate for the weight of the IVAN links, which fluctuates due to the economic growth and economic crisis that occurred in those 15 years. In Fig 1, each cell colored on the number of communities with more than 240 nodes which is 10% of the total nodes in the IVANs. We named these communities major communities. The remaining number of links in 2014 is almost half of that in 2004–2009.

**Fig 2:**
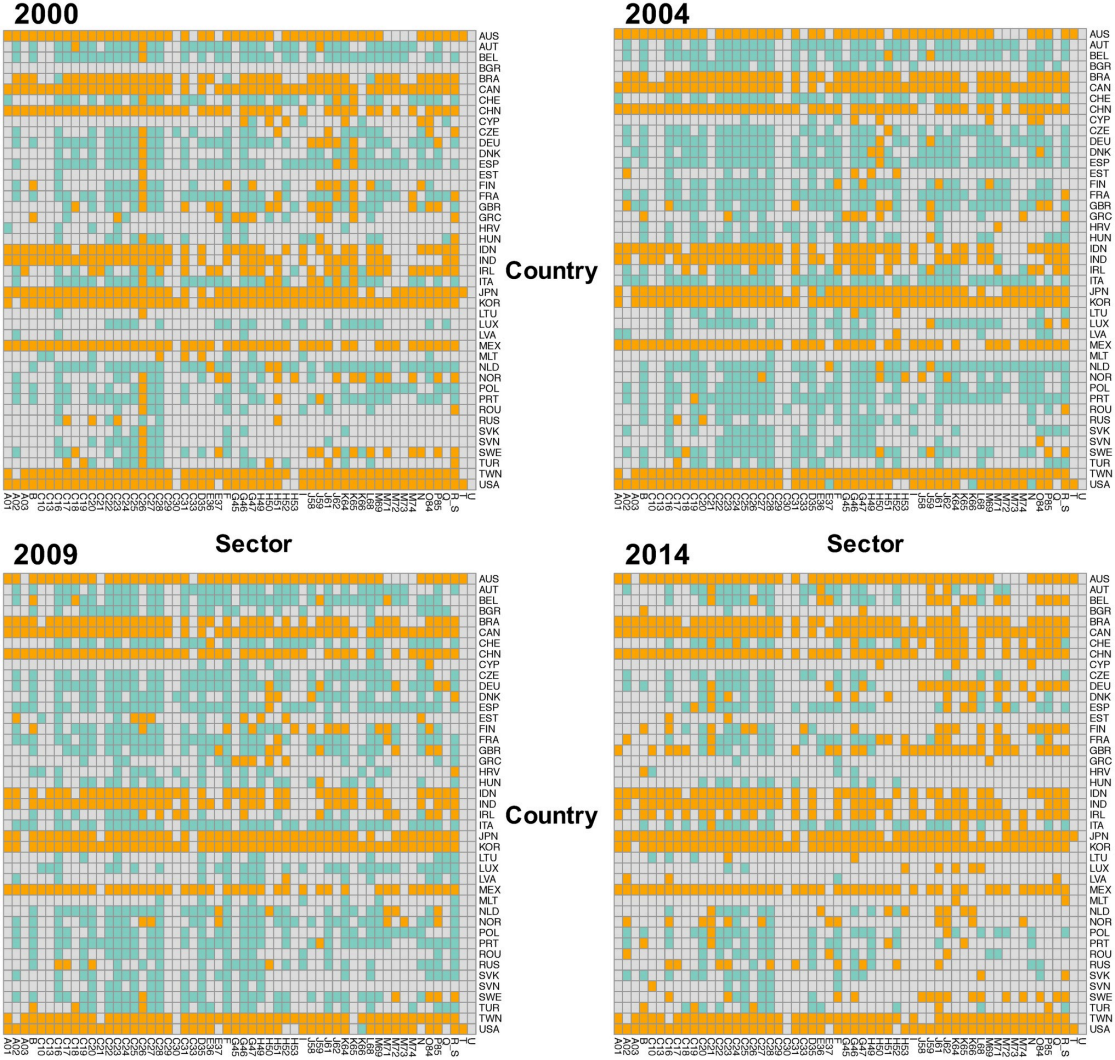
Community maps in 2000, 2004, 2009, and 2014. Each cell is a node of IVAN, the gray cells are the nodes that do not belong to any of two communities, while the other colors indicate community. The green ones belong to the community dominated by sectors of European countries and the orange ones to the community dominated by sectors of the Pacific Rim countries. The regional classification is in S1 Table.

By using the abovementioned threshold values, we can detect the communities from the IVANs as shown in Fig 2 which shows four years’ heat maps and represents each cell as a node of the IVANs. Fig 2 illustrates the results of the red-bordered cells of Fig 1. In the maps, the separated communities are indicated by different colors; the gray cells are the nodes that do not belong to any of those two communities. The figure shows orange horizontal stripes, including most of the sectors in countries such as Australia, Brazil, and Canada. The green nodes include the sectors A01: agriculture, C29: manufacture of motor vehicles, and C30: manufacture of other transport equipment of mainly European countries. The gray area includes many other sectors in Europe, especially in small countries. We define the two major communities as European and Pacific Rim communities, which are the automatically detected communities with Infomap mainly formed by nodes from Europe and Pacific Rim, respectively.

**Fig 3:**
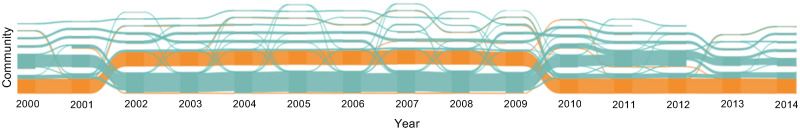
Sankey diagram of communities colored by actual regions where nodes are situated. Communities in each year are ordered by their size, from the largest to the smallest, from the bottom to the top. Green and orange in Fig 3 do not mean regional communities but mean the regions in which the node is positioned. The crossings of the major communities in 2001–2002 and 2009–2010 mean changes in the order of their size. Their classification of them is in S1 Table. The two bottom communities are major regional communities, but there had been other small European communities over the years.

The results of community detection 15 years are shown in Fig 3 as a Sankey diagram, where orange represents the Pacific Rim sectors, and green represents the European sectors (nodes) (the details of the classification are in S1 Table). In Fig 1, we illustrate the number of major communities, which include more than 10% of all (2408) nodes of IVAN. However, Fig 3 also represents minor communities that include less than 10% of all nodes. The relatively larger two communities at the bottom are the regional communities.

### Observed structural characteristics

Table 2 shows the structural features for 2000, 2008, and 2014. The densities and cluster coefficients show that the IVANs are dense, and the reciprocity shows that more than 97% of the links are mutual. The density of the IVANs that are cut at the threshold in Fig 1 is reduced by about 82%. In terms of reciprocities, the cut IVANs are about 20% of the remaining links that are cut. This indicates that the IVAN has many asymmetric mutualities with large values in only one direction. In the two local communities, the cluster coefficients and reciprocities are at the same level as those of the IVANs, although the densities are about 10% higher than those of the entire IVANs.

**Table 2:** Structural characteristics of IVANs, IVANs cut with the threshold, IVANs in the regional communities: Europe and the Pacific Rim.

	2000				2008				2014			
	IVAN	Cut IVAN	Europe	Pacific Rim	IVAN	Cut IVAN	Europe	Pacific Rim	IVAN	Cut IVAN	Europe	Pacific Rim
Density	0.831	0.0174	0.942	0.921	0.841	0.0184	0.955	0.925	0.831	0.0112	0.945	0.929
Reciprocity	0.972	0.231	0.987	0.976	0.982	0.192	0.990	0.987	0.974	0.169	0.993	0.975
Clustering coefficient	0.976	0.321	0.953	0.941	0.976	0.319	0.963	0.934	0.976	0.278	0.950	0.950
Diameter	2	5	2	2	2	4	2	2	2	6	2	2
Average path length	1.023	2.474	1.046	1.056	1.023	2.55	1.035	1.063	1.023	2.656	1.048	1.048
Average betweenness*	51.70	2644	24.62	37.14	51.72	3053	28.99	37.37	51.64	2835	14.32	35.82
Assortativity	-0.0167	-0.436	-0.0394	-0.0304	-0.0179	-0.427	-0.0294	-0.0305	-0.0170	-0.424	-0.0505	-0.0304
Average in-degree*	2196	71.68	516.4	622	2198	71	788	556.8	2193	52.59	281.7	715.3
Average out-degree*	2143	68.71	509.7	610.7	2164	65.5	780.3	553	2147	45.61	279.8	703

* These averages were calculated for nonzero values.

Each IVAN has a diameter of 2, which means that any sector in any country is connected to all sectors through one sector in another country. The minimum diameter is two because links to sectors in the same country are not included, but since the average path length is close to 1, most sectors are directly connected. In the cut IVANs, the diameter is about 5, and the average betweenness is more than 50 times larger than that of the original IVANs because the diameter and average path length are long, and a particular node mediates many shortest paths. In the regional communities, the average path length is slightly longer, and the betweenness is roughly half of the original IVANs.

The number of nodes in the IVANs is 2,408, and the average in-degree and out-degree value are over 2,100. Since most of the sectors are connected, the assortativity is close to zero. In the cut IVANs, the degrees and assortativity are around 70 and -0.43, respectively, indicating that sectors with degree differences are connected by high values flows. In the regional communities, the degree varies according to the size of the detected regional community, and the assortativity is negative, close to zero.

Table 2 includes three points: 1) no much difference in characteristics of IVANs between around 2006 (the lowest threshold year) and 2014 (the highest threshold year) are 2) influences of threshold on IVAN in each year 3) regional communities with similar characteristics, except for average betweenness and degrees, which are affected by size in each year. For the first point, the features of IVANs in 2014 and 2000 are almost the same, and the IVANs in 2008 have relatively higher density and assortativity. For the second point, we mentioned above the differences between original IVAN and cut IVAN. For the third point, most characteristics of the regional communities were similar, but there were significant differences in degrees that reflect the size of the community, especially in 2014.

**Fig 4:**
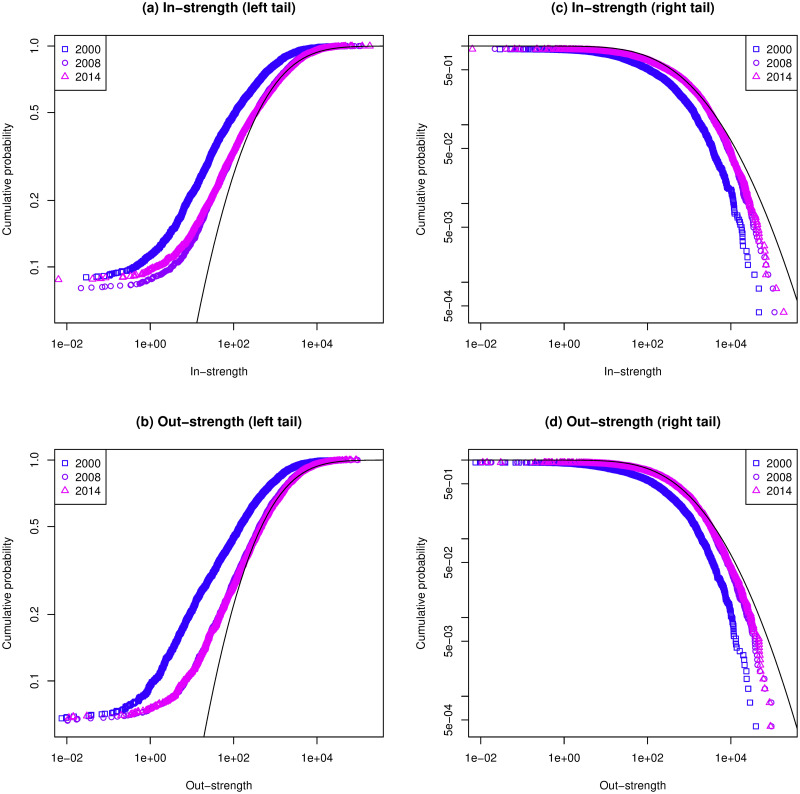
Cumulative probability of strength of IVAN in 2000, 2008, and 2014. The black lines are plots of the cumulative density function of the log-normal distribution whose *μ* and *σ* are equal to the average and the standard deviation, respectively, of the logarithm of strength distribution in 2014. Their right tails are almost fitted, but the left tails are not, especially at the bottom.

Fig 4 shows a wide strength distribution of the IVAN’s. The distribution of Fig 4 partly fits a log-normal distribution, especially in the right tail. The probability density function of a log-normal distribution *p*(*x*) for *x* > 0 is:

$$\begin{array}{c} p\left(x\right)=\frac{1}{\sqrt{2\pi }\sigma x}\exp[-\frac{{\left(\log x-\mu \right)}^{2}}{2\sigma^{2}}], \end{array}$$ (5)

where *μ* and *σ* are the mean and standard deviation of the logarithm, respectively. As seen in Fig 4, the mean and standard deviation of the logarithm of strength distribution in 2014 are *μ* = 5.959 and *σ* = 2.129 in in-strength, and *μ* = 6.146 and *σ* = 2.001 in out-strength, respectively.

We examined whether the difference in the threshold values before and after the economic crisis in Fig 1 was due to the difference in the network features. Fig 4 shows that the strength distributions in 2008 and 2014 are almost equal. Therefore, this indicates that there is no significant change in the IVAN as a network structure, which does not appear in the features used in this study.

### Potential and circular relationships in two regional communities

**Fig 5:**
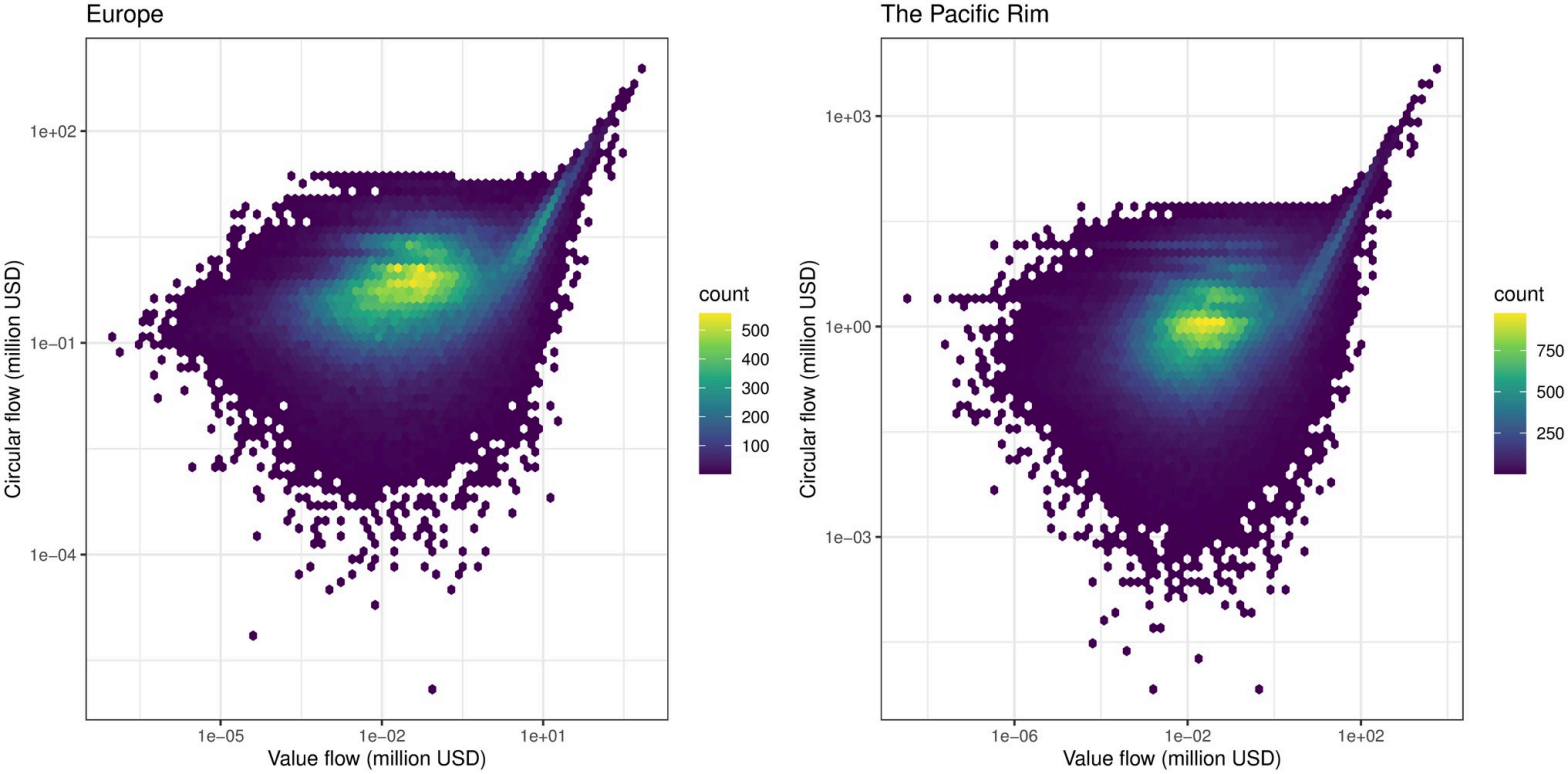
Scatter plot of correlation between circular flow and value flow of two regional communities in 2000. The larger circular flows are in proportion to the original value flows, but the lower flows are disarranged.

We applied Helmholtz–Hodge decomposition to the two regional communities detected by Infomap. The size of the circular flow component and the value of the IVAN link (value flow) are well correlated in the range where the value flow is greater than 100 million USD, as shown in Fig 5. In the economic integration index, the amount of circular flow is divided by the GVAN weights; thus, the economic scale does not appear directly.

The potential and circular relationships in European and the Pacific Rim communities can be analyzed from the international and intersectoral perspectives by aggregating nodes into countries and sectors. As Fig 2 illustrates, some countries were present with a smaller number of sectors in each community.

#### European community

Tables 3–8 show the breakdown of the potential and circular relationships in Europe, which is obtained from the Helmholtz–Hodge decomposition. In terms of countries, the value potential is mainly high in Russia, Norway, and Germany (Table 3) and low in France, Spain, and Italy (Table 4). From 2003 to 2012, Russia and Norway mainly have the first and second highest potential, respectively. In 2001, 2002, and 2010, with changes in the ranking of the communities in Fig 3, Germany shows the highest potential. In 2000 and 2001, 2010, and 2013, when the European community was relatively small (Fig 3), Russia had not been in Table 3. In addition, Belgium, Switzerland, and the Netherlands were the other high-potential countries that were ranked repeatedly in Table 3. Non-European countries, such as the United States, had also been ranked because of specific industries in the European Community. The United States was ranked as a high-potential country in 2002–2005, before the economic crisis, and China was ranked as a high-potential country in the European Community in 2009–2010, during the economic crisis. As for low-potential countries (Table 4), basically France had the lowest potential, and Spain had the lowest potential in 2002 and 2003. Table 4 also represented Italy, Portugal, and Turkey as low-potential countries in half of the years, and the United Kingdom as a low-potential country after 2007. Tables 3 and 4 show that Germany is both a high-potential (in 2001–2007, 2009, 2010, 2014) and low-potential country (in 2008, 2011–2013).

**Table 3:** Five highest-potential countries in European community.

Rank	2000	2001	2002	2003	2004	2005	2006	2007	2008	2009	2010	2011	2012	2013	2014
1	NLD	DEU	DEU	RUS	RUS	RUS	RUS	RUS	RUS	RUS	DEU	RUS	RUS	POL	DEU
2	DEU	BEL	RUS	NOR	NOR	NOR	NOR	NOR	NOR	NOR	GBR	NOR	NOR	ITA	RUS
3	CHE	CHE	NOR	DEU	DEU	DEU	DEU	DEU	NLD	DEU	BEL	NLD	NLD	CZE	NLD
4	BEL	NLD	USA	USA	USA	USA	NLD	NLD	CHE	CHN	CHN	IRL	DNK	AUT	GBR
5	NOR	FIN	NLD	NLD	BEL	BEL	USA	BEL	AUT	BEL	AUT	CYP	USA	NLD	HUN

**Table 4:** Five lowest-potential countries in European community.

Rank	2000	2001	2002	2003	2004	2005	2006	2007	2008	2009	2010	2011	2012	2013	2014
1	FRA	FRA	ESP	ESP	FRA	FRA	FRA	ESP	FRA	FRA	FRA	FRA	FRA	FRA	FRA
2	ESP	ESP	ITA	FRA	ESP	ESP	ESP	FRA	ESP	ITA	ITA	DEU	DEU	BEL	CHE
3	ITA	DNK	FRA	ITA	ITA	ITA	ITA	GBR	DEU	ESP	ESP	ITA	GBR	GBR	DNK
4	PRT	PRT	POL	POL	HUN	TUR	TUR	TUR	TUR	GBR	LUX	GBR	TUR	RUS	ESP
5	DNK	POL	PRT	HUN	POL	HUN	POL	ITA	POL	LUX	DNK	POL	POL	DEU	TUR

**Table 5:** Five highest-potential sectors in European community.

Rank	2000	2001	2002	2003	2004	2005	2006	2007	2008	2009	2010	2011	2012	2013	2014
1	M69	C24	B	B	B	B	B	B	B	B	M69	B	B	C25	G46
2	C24	N	N	N	N	N	N	C24	C24	M69	G46	G46	G46	C24	C24
3	N	M69	M69	M69	M69	C24	C24	N	G46	N	N	N	N	C22	C25
4	C20	C20	C24	C24	C24	M69	M69	G46	M69	G46	C24	C24	M69	G46	C20
5	K64	C25	C20	C20	C20	G46	G46	M69	H49	C24	C20	H49	C24	C27	C22

B: mining and quarrying; C20: manufacture of chemicals and chemical products; C22: manufacture of rubber and plastic products; C24: manufacture of basic metals; C25: manufacture of fabricated metal products except machinery and equipment; C27: manufacture of electrical equipment; G46: wholesale trade except trade of motor vehicles and motorcycles; H49: land transport and transport via pipelines; K64: financial service activities except insurance and pension funding; M69: legal and accounting activities; activities of head offices, and management consultancy activities; N: administrative and support service activities.

**Table 6:** Five lowest-potential sectors in European community.

Rank	2000	2001	2002	2003	2004	2005	2006	2007	2008	2009	2010	2011	2012	2013	2014
1	F	F	F	F	F	F	F	F	F	F	F	F	F	F	F
2	C28	C28	C28	C28	C19	C19	C19	C29	C29	C19	C28	C19	C19	C29	C28
3	C19	O84	O84	O84	C28	C28	C28	C19	C19	C28	O84	C28	C28	C28	O84
4	O84	C31	C19	C19	O84	O84	O84	C28	C28	O84	G47	D35	D35	O84	G47
5	C31	L68	L68	L68	L68	R_S	R_S	O84	O84	L68	C31	O84	O84	R_S	C33

C19: manufacture of coke and refined petroleum products; C28: manufacture of machinery and equipment n.e.c.; C29: manufacture of motor vehicles, and trailers, and semi-trailers; C31: manufacture of furniture, and other manufacturing; C33: repair and installation of machinery and equipment; D35: electricity, gas, steam and air conditioning supply; F: construction; G47: retail trade except that of motor vehicles and motorcycles; L68: real estate activities; O84: public administration and defense, and compulsory social security; R_S: other service activities.

**Table 7:** Five-highest circulated countries in European community.

Rank	2000	2001	2002	2003	2004	2005	2006	2007	2008	2009	2010	2011	2012	2013	2014
1	DEU	DEU	DEU	DEU	DEU	DEU	DEU	DEU	DEU	DEU	DEU	DEU	DEU	DEU	DEU
2	FRA	FRA	GBR	FRA	FRA	FRA	FRA	FRA	RUS	FRA	FRA	RUS	RUS	FRA	FRA
3	NLD	ITA	FRA	ITA	GBR	GBR	GBR	ITA	FRA	RUS	ITA	NOR	GBR	ITA	ITA
4	ITA	ESP	ITA	GBR	ITA	ITA	RUS	RUS	ITA	GBR	ESP	FRA	NOR	ESP	POL
5	ESP	GBR	ESP	ESP	NLD	RUS	ITA	GBR	NOR	ITA	NLD	GBR	FRA	POL	AUT

**Table 8:** Five-highest circulated sectors in European community.

Rank	2000	2001	2002	2003	2004	2005	2006	2007	2008	2009	2010	2011	2012	2013	2014
1	F	F	F	F	F	F	F	F	B	F	F	B	B	C29	F
2	C28	C28	B	B	B	B	B	B	F	B	C28	F	F	F	C28
3	G46	G46	N	N	C19	C19	C19	C29	C19	C19	G46	C19	C19	C28	G46
4	K64	C20	G46	G46	N	G46	G46	C19	C29	G46	M69	G46	D35	C25	C25
5	C20	C24	C28	C28	G46	C28	C28	G46	G46	D35	K64	D35	G46	C24	C20

B: mining and quarrying; C19: manufacture of coke and refined petroleum products; C20: manufacture of chemicals and chemical products; C24: manufacture of basic metals; C25: manufacture of fabricated metal products, except machinery and equipment; C28: manufacture of machinery and equipment n.e.c.; C29: manufacture of motor vehicles, and trailers and semi-trailers; D35: electricity, gas, steam and air conditioning supply; F: construction; G46: wholesale trade except that of motor vehicles and motorcycles; K64: financial service activities except insurance and pension funding; M69: legal and accounting activities, activities of head offices, and management consultancy activities; N: administrative and support service activities.

In general, Russia was ranked first in the high-potential table (Table 3) when sector B: mining and quarrying was ranked first (Table 5). The other highest-potential sectors in Europe were M69: legal, accounting, and consultancy activities in 2000 and 2010; C24: manufacture of basic metals in 2001; C25: manufacture of fabricated metal products, except machinery and equipment in 2013; and G46: wholesale trade except that of motor vehicles and motorcycles in 2014. In addition, sectors N: administrative and support service activities and C20: manufacture of chemicals and chemical products were often ranked high. In terms of low potential (Tables 4 and 6), sector F: construction had been ranked first throughout the 15 years, followed by sectors C28: manufacture of machinery and equipment, C19: manufacture of coke and refined petroleum products, C29: manufacture of motor vehicles, which occupy the second and subsequent positions; O84: public administration and defense, and compulsory social security, which appears in the third and subsequent positions; and L68: real estate activities, which often appears in the fifth position.

According to the amount of circulation (Table 7), Germany had been the top country for the 15 years, followed by France, the United Kingdom, Italy, and Russia. In terms of sectoral ranking (Table 8), sector F: construction is consistently ranked first until 2010. In 2011 and 2012, sector B: mining and quarrying, which often ranked second, was ranked first. In 2013, sector C29: manufacture of the motor vehicle was first. Russia was ranked second in the year that sector B: mining and quarrying was ranked first. Throughout the whole years, F: construction was first, followed by B: mining and quarrying, C28: manufacture of machinery and equipment, C19: manufacture of coke and refined petroleum products, and G46: wholesale trade except for that of motor vehicles and motorcycles.

**Fig 6:**
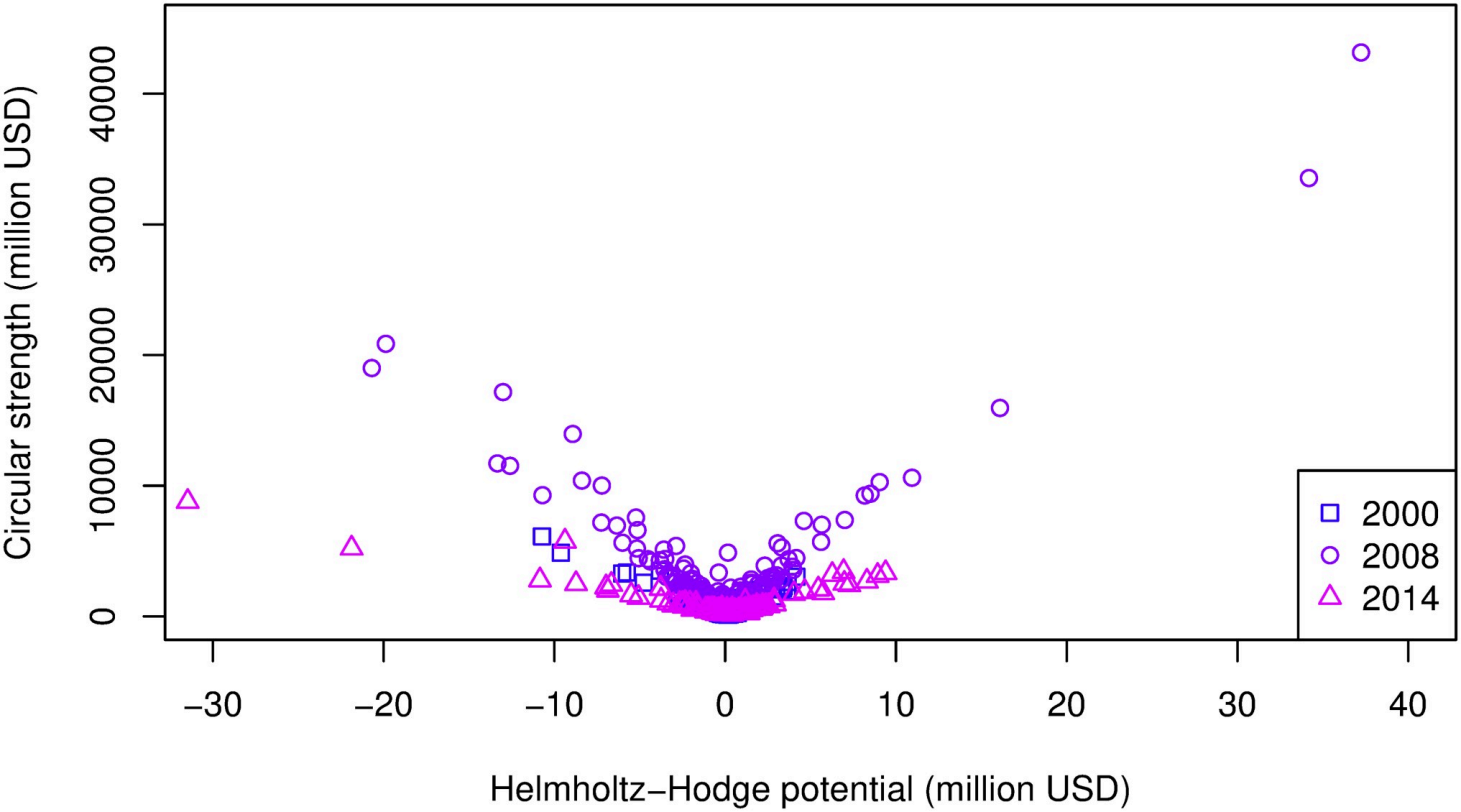
Scatter plot of correlation between circular strength and Helmholtz–Hodge potential in European community in 2000, 2008, and 2014. Each year shows a V-shaped pattern, but the inclination is changed (sharpest in 2008 and gentlest in 2014).

For both countries and sectors, those ranked higher or lower in terms of potential are also greater in terms of circular strength, which is the strength of the circular network decomposed from an IVAN. The relationship between the circular strength and the Helmholtz–Hodge potential is shown in Fig 6. The relationship is V-shaped; in other words, the larger the absolute value of the potential, the higher the circular strength.

#### The Pacific Rim community

The characteristics of the potential and circular flows of the IVANs in the Pacific Rim are shown in Tables 9–14. Japan, which had the maximum potential in 2000 and 2002, had been out of the ranking since 2012; Canada showed the maximum potential from 2003 to 2007, and Australia had been the highest-potential country since then (Table 9). Indonesia, Taiwan, and India were also among the five highest-potential countries. In terms of low potential, Mexico was the top-ranked country until 2002, the United States from 2004 to 2006, and China in 2003 and 2007 to 2013. In addition, India, the United Kingdom, and France appeared in Table 10 numerous times.

**Table 9:** Five highest-potential countries in the Pacific Rim community.

Rank	2000	2001	2002	2003	2004	2005	2006	2007	2008	2009	2010	2011	2012	2013	2014
1	JPN	CAN	JPN	CAN	CAN	CAN	CAN	CAN	AUS	AUS	AUS	AUS	AUS	AUS	AUS
2	CAN	JPN	CAN	JPN	JPN	JPN	AUS	AUS	CAN	CAN	JPN	CAN	CAN	CAN	CAN
3	AUS	AUS	AUS	AUS	AUS	AUS	JPN	JPN	IDN	JPN	CAN	IDN	IDN	TWN	TWN
4	IDN	IDN	TWN	IDN	IDN	IDN	IDN	TWN	TWN	TWN	IDN	JPN	TWN	RUS	KOR
5	TWN	TWN	IDN	TWN	KOR	TWN	TWN	IDN	BRA	IDN	TWN	TWN	BRA	IDN	DEU

**Table 10:** Five lowest-potential countries in the Pacific Rim community.

Rank	2000	2001	2002	2003	2004	2005	2006	2007	2008	2009	2010	2011	2012	2013	2014
1	MEX	MEX	MEX	CHN	USA	USA	USA	CHN	CHN	CHN	CHN	CHN	CHN	CHN	USA
2	CHN	CHN	USA	USA	CHN	CHN	CHN	USA	USA	MEX	MEX	IND	USA	USA	CHN
3	USA	GBR	CHN	MEX	MEX	MEX	MEX	MEX	MEX	IND	GBR	MEX	IND	MEX	MEX
4	GBR	IND	IND	IND	GBR	IRL	IRL	IND	KOR	GBR	IND	KOR	MEX	IDN	IND
5	IND	USA	FRA	FRA	IND	IND	GBR	HUN	HUN	DEU	KOR	USA	FRA	GBR	GBR

**Table 11:** Five highest-potential sectors in the Pacific Rim community.

Rank	2000	2001	2002	2003	2004	2005	2006	2007	2008	2009	2010	2011	2012	2013	2014
1	B	B	B	B	B	B	B	B	B	B	B	B	B	B	B
2	C20	C20	C20	C20	C20	C24	C24	C24	C24	C24	C20	C24	C24	G46	G46
3	C24	C24	C24	C24	C24	C20	C20	G46	G46	C20	G46	C20	G46	C24	C24
4	G46	G46	G46	G46	G46	G46	G46	C20	C20	G46	C24	G46	C20	C20	C20
5	N	N	C25	K64	K64	K64	K64	K64	K64	K64	N	N	K64	K64	K64

B: mining and quarrying; C20: manufacture of chemicals and chemical products; C24: manufacture of basic metals; C25: manufacture of fabricated metal products except machinery and equipment; G46: wholesale trade except that of motor vehicles and motorcycles; K64: financial service activities except insurance and pension funding; N: administrative and support service activities.

**Table 12:** Five lowest-potential sectors in the Pacific Rim community.

Rank	2000	2001	2002	2003	2004	2005	2006	2007	2008	2009	2010	2011	2012	2013	2014
1	F	F	F	F	F	F	F	F	F	F	F	F	F	F	F
2	O84	O84	O84	O84	O84	O84	O84	O84	O84	O84	O84	O84	O84	Q	O84
3	C29	C29	C29	C29	C29	C29	C29	C29	C29	C29	Q	Q	Q	O84	Q
4	C10	Q	C10	C10	C10	C10	C10	C10	C10	C10	C29	C29	C29	C29	C29
5	Q	C10	Q	Q	Q	Q	Q	Q	Q	Q	C10	C10	C10	C10	C10

C10: manufacture of food products, beverages and tobacco products; C29: manufacture of motor vehicles, and trailers and semi-trailers; F: construction; O84: public administration and defense, and compulsory social security; Q: human health and social work activities.

**Table 13:** Five highest-circulated countries in the Pacific Rim community.

Rank	2000	2001	2002	2003	2004	2005	2006	2007	2008	2009	2010	2011	2012	2013	2014
1	USA	USA	USA	USA	USA	USA	USA	USA	USA	USA	USA	USA	USA	USA	USA
2	JPN	CAN	CAN	CAN	CAN	CAN	CAN	CHN	CHN	CHN	CHN	CHN	CHN	CHN	CHN
3	CAN	JPN	JPN	JPN	JPN	CHN	CHN	CAN	CAN	CAN	CAN	CAN	CAN	CAN	CAN
4	MEX	MEX	MEX	CHN	CHN	JPN	JPN	JPN	JPN	JPN	JPN	JPN	JPN	JPN	JPN
5	CHN	CHN	CHN	MEX	MEX	MEX	MEX	MEX	MEX	MEX	MEX	MEX	MEX	MEX	MEX

**Table 14:** Five highest-circulated sectors in the Pacific Rim community.

Rank	2000	2001	2002	2003	2004	2005	2006	2007	2008	2009	2010	2011	2012	2013	2014
1	C26	F	F	F	F	F	F	C26	B	F	F	B	B	F	F
2	F	C26	C26	C26	B	B	B	F	C26	B	B	F	F	B	B
3	B	B	B	B	C26	C26	C26	B	F	C26	C26	C26	C26	C26	C26
4	C29	O84	C29	O84	O84	C29	O84	O84	O84	O84	O84	O84	O84	O84	O84
5	O84	C29	O84	C29	C29	O84	C29	C29	C29	C29	C29	Q	Q	Q	Q

B: mining and quarrying; C26: manufacture of computer, electronic, and optical products; C29: manufacture of motor vehicles, and trailers and semi-trailers; F: construction; O84: public administration and defense, and compulsory social security; Q: human health and social work activities.

In terms of sectoral potential (Tables 11 and 12), as in Europe, B: mining and quarrying was the highest-potential sector, and F: construction was the lowest-potential sector. The relationship was more stable than that in Europe and remained unchanged over the 15 years. In addition, as shown in Table 11, sectors B: mining and quarrying, C20: manufacture of chemicals and chemical products, C24: manufacture of basic metals, G46: wholesale trade, K64: financial service activities, and N: administrative and support service activities were included in the high-potential sectors. Sectors F: construction; O84: public administration and defense, and compulsory social security; C29: manufacture of motor vehicles; C10: manufacture of food products, beverages, and tobacco products; and Q: human health and social work activities were among the lowest-potential sectors until 2009 except 2001. Since 2010, Q: human health and social work activities had risen to higher than third place.

In terms of circulation (Tables 13 and 14), the United States had been consistently in the first place, followed by Japan, Canada, and China since 2007. Since 2005, Japan had been in the fourth place, followed by Mexico. By contrast, the sector rankings in Table 14 were not as consistent as those of the countries. In 2000 and 2007, C26: manufacture of computer, electronic, and optical products was ranked first; in 2008 and 2011, B: mining and quarrying was ranked first. These three sectors remained in the top three positions over the 15 years. They were followed by C29: manufacture of motor vehicles and O84: public administration and defense, and compulsory social security until 2009, and by O84: public administration and defense, and compulsory social security and Q: human health and social work activities since 2010.

**Fig 7:**
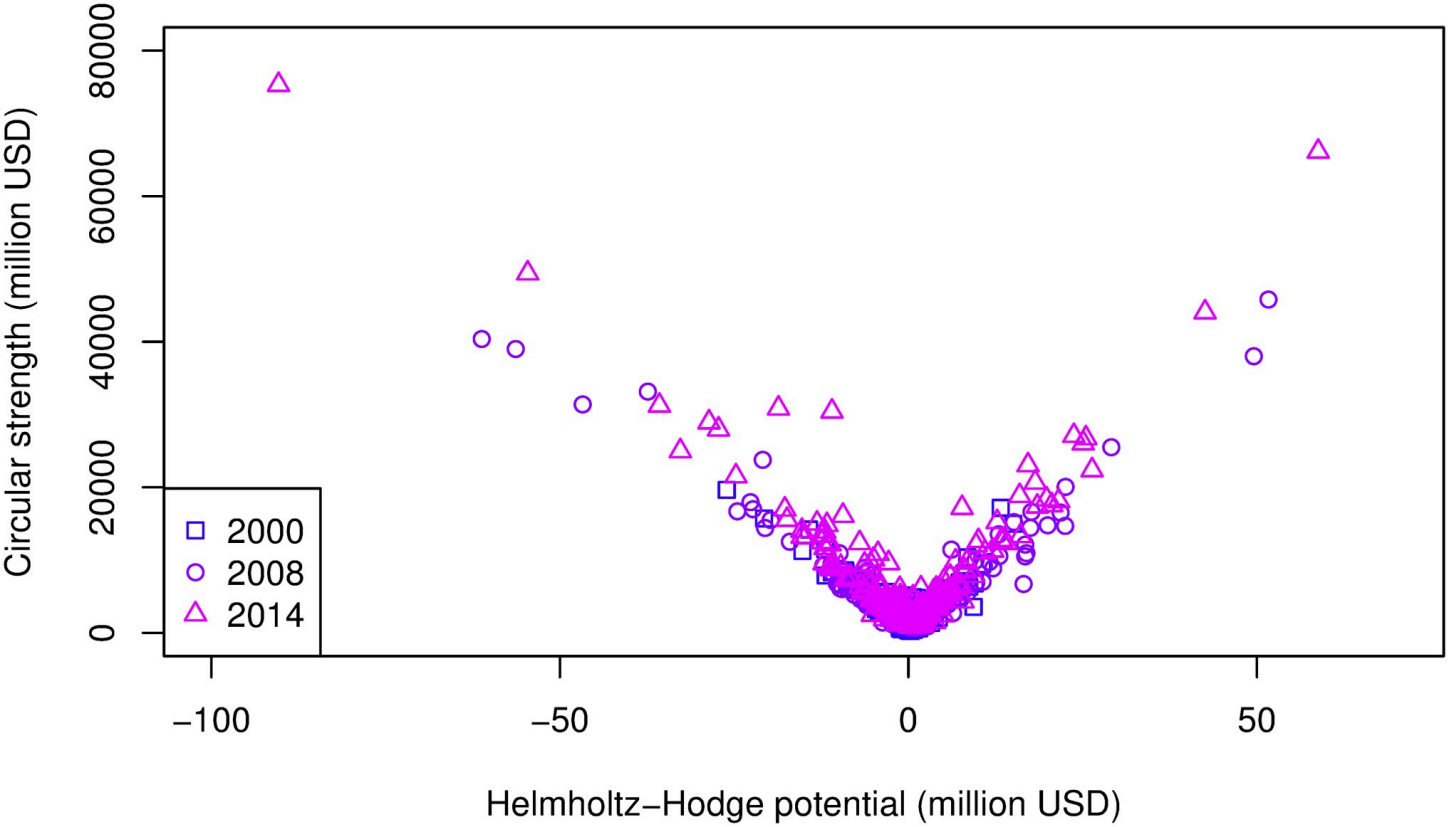
Scatter plot of correlation between circular strength and Helmholtz-Hodge potential in the Pacific Rim community in 2000, 2008, and 2014. The years show the similar inclinations of the V shape.

As in European community, the high- or low-potential countries and sectors in the Pacific Rim community were high in circular strength. The relationship between the circular strength and the Helmholtz–Hodge potential is shown in Fig 7. The V-shaped relationship between them is the same as that in European community. However, there is a difference in Tables 2–14. Sector C26: manufacture of computer, electronic, and optical products did not appear in Tables 11 and 12, which indicates a large value-added circulation in the midstream of the Pacific Rim’s value flow, although all high-circular-strength sectors in Europe were also in the value-added potential ranking.

### Economic integration index

**Fig 8:**
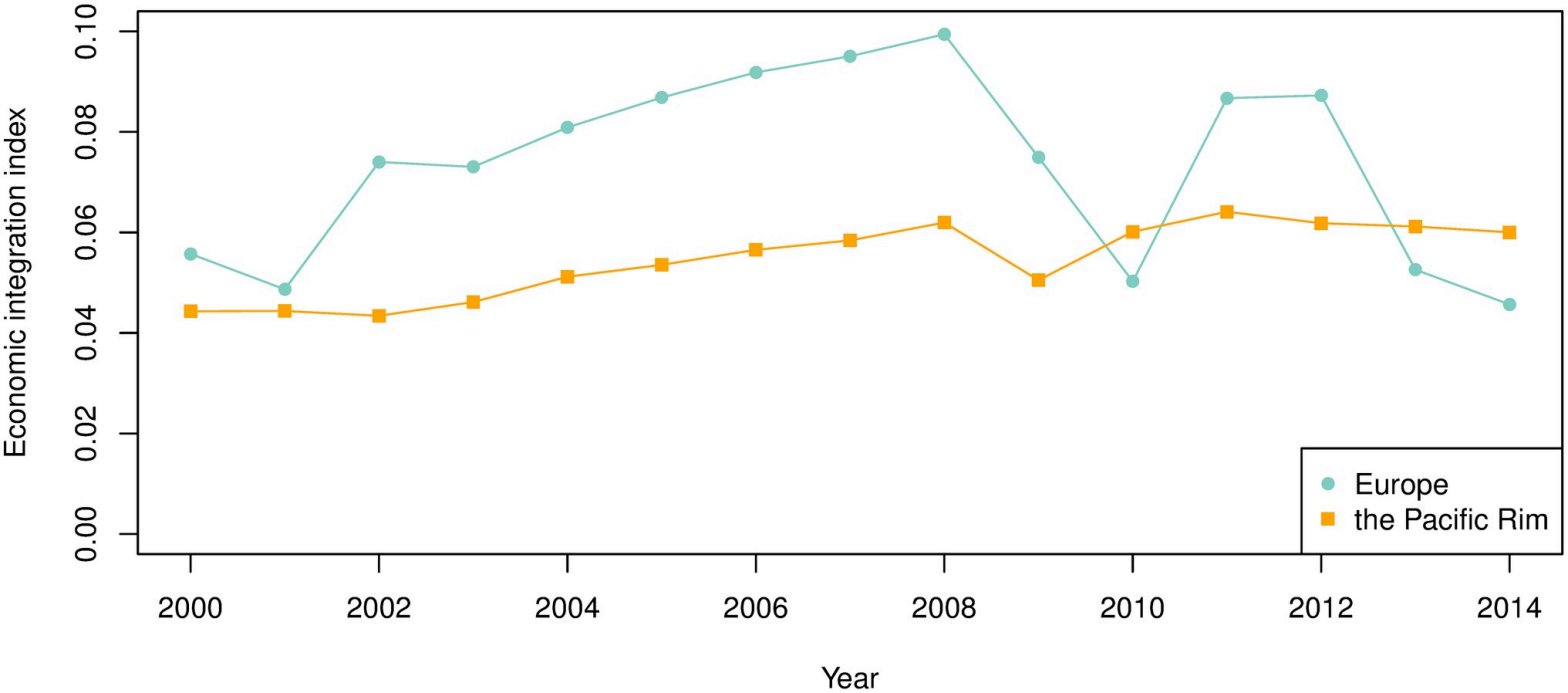
Economic integration estimation of the two regional communities in 2000–2014.

The results of applying the economic integration index to the two regional communities are shown in Fig 8. The Pacific Rim community showed a stable and upward trend for economic integration, while European community showed a higher but more unstable integration level. In particular, there was a large decline in the level of integration in 2009 and 2010, after the economic crisis, while the Pacific Rim community was slightly affected in 2009.

**Fig 9:**
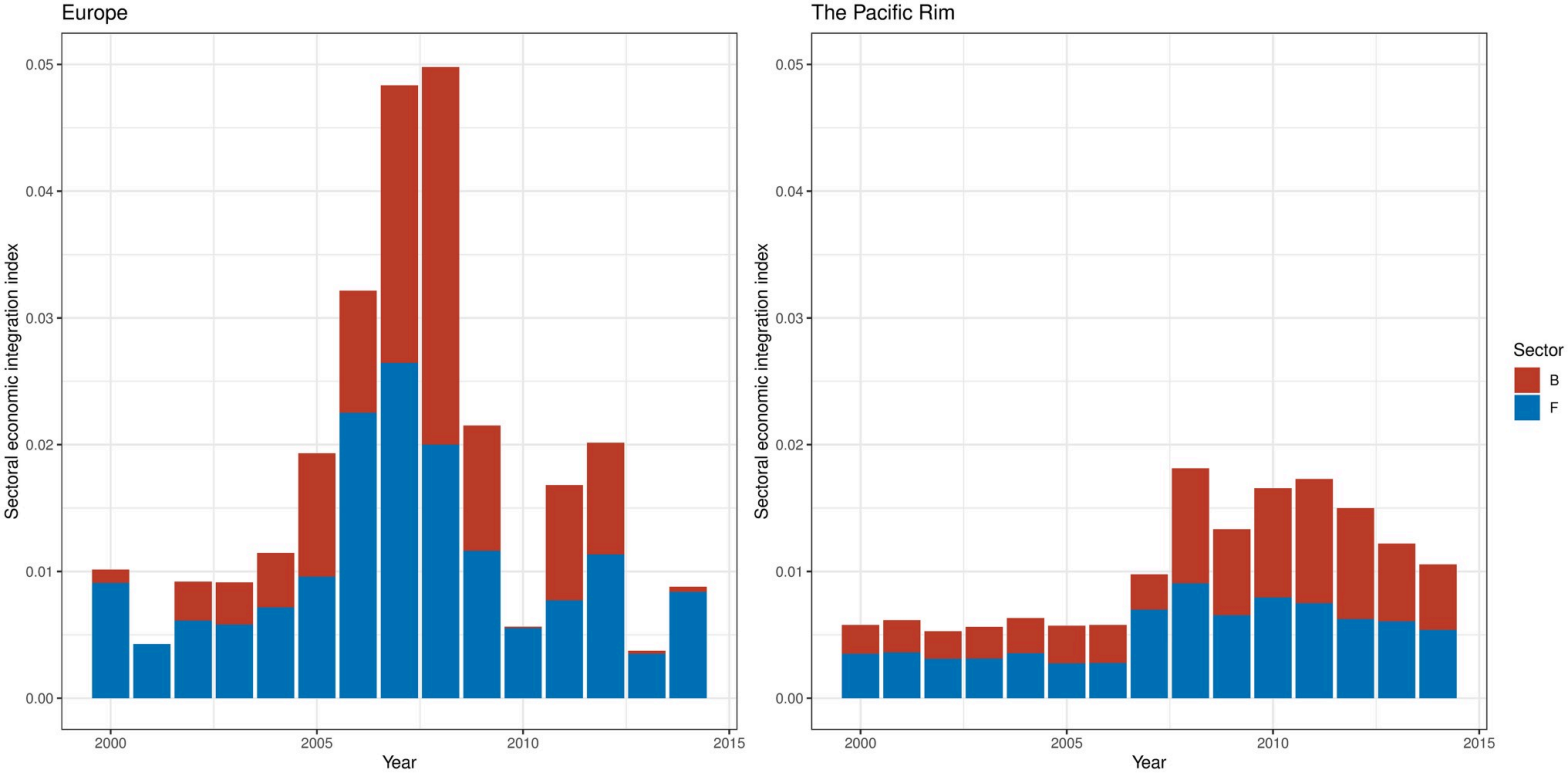
Sectoral economic integration index of mining and quarrying, and construction in two regional communities. B: mining and quarrying, F: construction.

**Fig 10:**
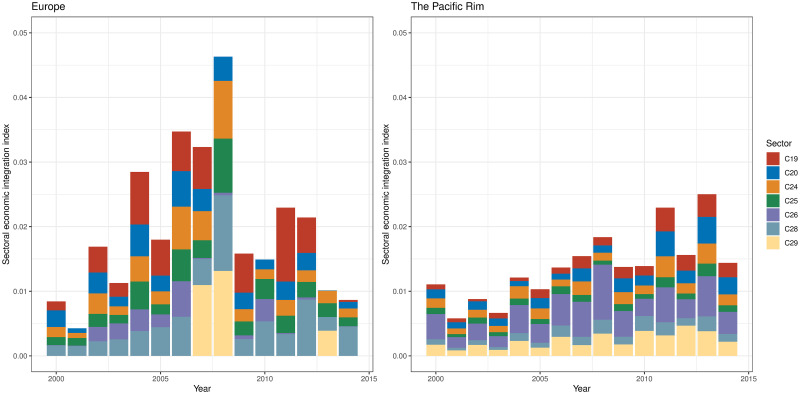
Sectoral economic integration index of manufacture in two regional communities. C19: manufacture of coke and refined petroleum products; C20: manufacture of chemicals and chemical products; C24: manufacture of basic metals, C25: manufacture of fabricated metal products except for machinery and equipment; C26: manufacture of computer, electronic, and optical products; C28: manufacture of machinery and equipment n.e.c.; C29: manufacture of motor vehicles, and trailers and semi-trailers.

**Fig 11:**
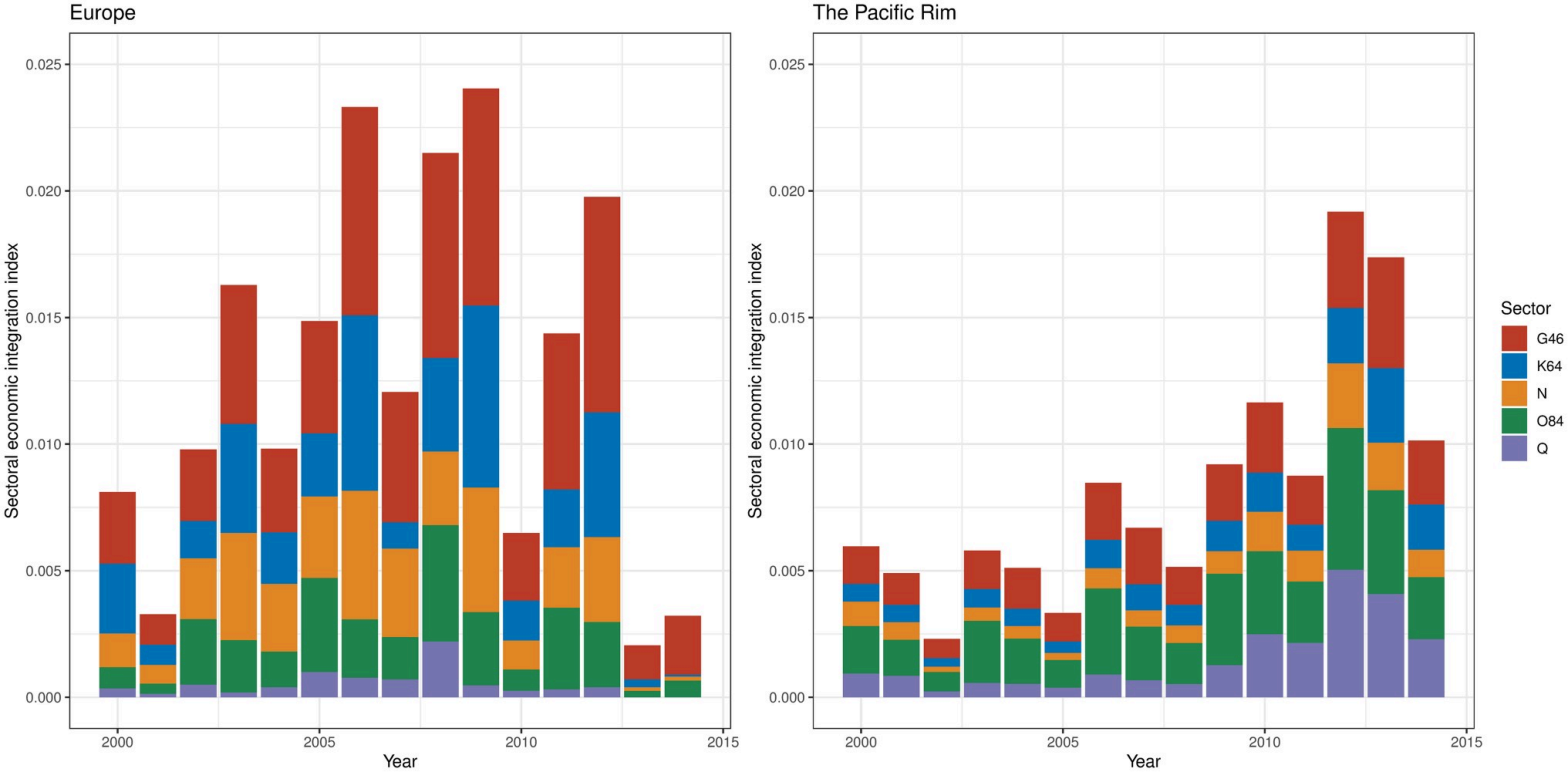
Sectoral economic integration index of other important sectors in two regional communities. G46: wholesale trade except motor vehicles and motorcycles; K64: financial service activities except for insurance and pension funding; N: administrative and support service activities; O84: public administration and defense, and compulsory social security; Q: human health and social work activities.

Figs 9–11 show the sector-wise economic integration indices, focusing on the sectors that showed high circulation in Tables 8 and 14. As illustrated in Fig 9, European community exhibited a fivefold increase in 2007 and 2008 (just before the economic crisis) in sector B: mining and quarrying and F: construction (the top-ranking sectors in terms of potential and circular flows) compared with 2000, then a sharp decline from 2009 to 2010 (below 2000 level). The low values in 2010, 2013, and 2014 were partly due to a decline in F: construction, but the circulation within B: mining and quarrying was almost zero. By contrast, in the Pacific Rim, these values were tripled between 2007 and 2008; they declined to begin 2011, but they remained above 2007 levels in 2014.

Fig 10 shows the sectoral economic integration indices of the manufacturing sectors ranked in Tables 8 and 14. The figure illustrates that the increase in circulation in the manufacturing sectors played a major role in Europe having the largest economic integration index in 2008. Sector C29: manufacture of motor vehicles made a significant contribution in 2007 and 2008. Sectors C24: manufacture of basic metals, C25: manufacture of fabricated metal products, and C29: manufacture of motor vehicles exhibited the highest values in 2008. The high value in 2008 can be seen from the fact that C24: manufacture of basic metals, C25: manufacture of fabricated metal products, and C29: manufacture of motor vehicles showed the highest values throughout the 15 years. As for the manufacturing sectors, C19: manufacture of coke and refined petroleum products disappeared from the European community when the economic integration index for B: mining and quarrying was nearly zero. On the contrary, in the Pacific Rim, the manufacturing sector steadily increased its contribution to economic integration. In the years with large values in the economic integration index, C19: manufacture of coke and refined petroleum products, C20: manufacture of chemicals and chemical products, and C29: manufacture of motor vehicles were large in sectoral indices.

Finally, Fig 11 shows the other sectors that are ranked three or more times in Tables 8 and 14. When European community showed a high degree of economic integration, the contributions of G46: wholesale trade, K64: financial service activities, and N: administrative and support service activities were large. The contributions of K64: financial service activities and N: administrative and support service activities became so small after 2013, when the level of integration was low, that they are barely visible. In the Pacific Rim, the contributions of G46: wholesale trade; O84: public administration and defense, and compulsory social security; and Q: human health and social work activities are large, although they tend to peak and decline every three years except for 2009 when the economic crisis occurred.

## Discussion

In the international trade networks used by Ikeda et al. [25], the communities are divided into industries (see S3 Table and S1 Fig); in original IVANs, the entire world is connected. Two buried communities—Europe and the Pacific Rim—were identified in the this paper by eliminating low flows with a threshold. However, since the IVAN included only 43 countries (excludes RoW), a more extensive international economic network, including other countries, may reveal more than two regional communities.

The variability between the threshold and the community detection results is also important. In this study, we did not set the threshold as a specific weight to see the change over the 15 studied years. This is because the weights of IVAN links increase or decrease depending on economic growth or crisis; thus, it was inappropriate to use the same value as the threshold throughout the 15 years. In addition, as seen in Fig 1, the results of the detected communities are unstable, even for close threshold values. The threshold value did not separate whether a single large community or two communities appeared; rather, the trend of the distribution changed. In this study, we used a resolution of every 500 links, but more detailed research is needed on the relationship between the number of remaining links and the number of detected communities. Thus, the threshold with the most remaining links was used for this analysis. The impact of this approach on the results is limited because the threshold was only applied for detecting concealed communities; it was not adopted for calculating the economic integration. With the threshold value we adopted, two communities with more than 240 nodes appeared, around 10% of the 2408 nodes included by the IVAN, and those communities were analyzed as regional communities. Therefore, the sector-specific European communities shown as gray nodes in Fig 2 and green small communities in Fig 3 were not treated as the regional community of Europe. In other words, the European community does not include sector-specific European communities because Europe has substantial value flows that have mainly the same type of sector, not different types of sectors.

In addition, the relationship between threshold and regional community detection shows a large difference before and after the economic crisis, but Table 2 or Fig 4 do not represent such a difference. More research is needed to understand how the threshold for the appearance of regional characteristics in IVAN can be interpreted from international trade, and whether it is related to the economic crisis.

In this study, we set a threshold for detecting major communities and calculated the degree of economic integration within the range found by the Infomap algorithm. Therefore, we interpreted that the economic integration of European communities is unstable because the industries that contribute the most to the level of economic integration are not continuously detected as European communities. However, since the results differ depending on the threshold value and the community detection algorithm, it remains to research how to set the appropriate threshold value when Infomap needs to detect communities in a real weighted directed network such as IVAN.

Originally, the smile curve [42] was viewed in terms of the production stage of a particular product or its relative position from a specific sector. In a broad sense, the smile curve in this study appears as a V-shaped curve when its relative position to the final goods of various sectors is considered as a whole. Not surprisingly, Helmholtz–Hodge decomposition shows a cross-country potential flow with high (low) potential in B: mining and quarrying (F: construction), which is in the upstream (downstream) stage of production. As seen from the circular relationship, the contributions of B: mining and quarrying and F: construction to international economic integration also indicate that the manufacture of each country depends on the resources and development demands (such as construction) of other countries.

**Fig 12:**
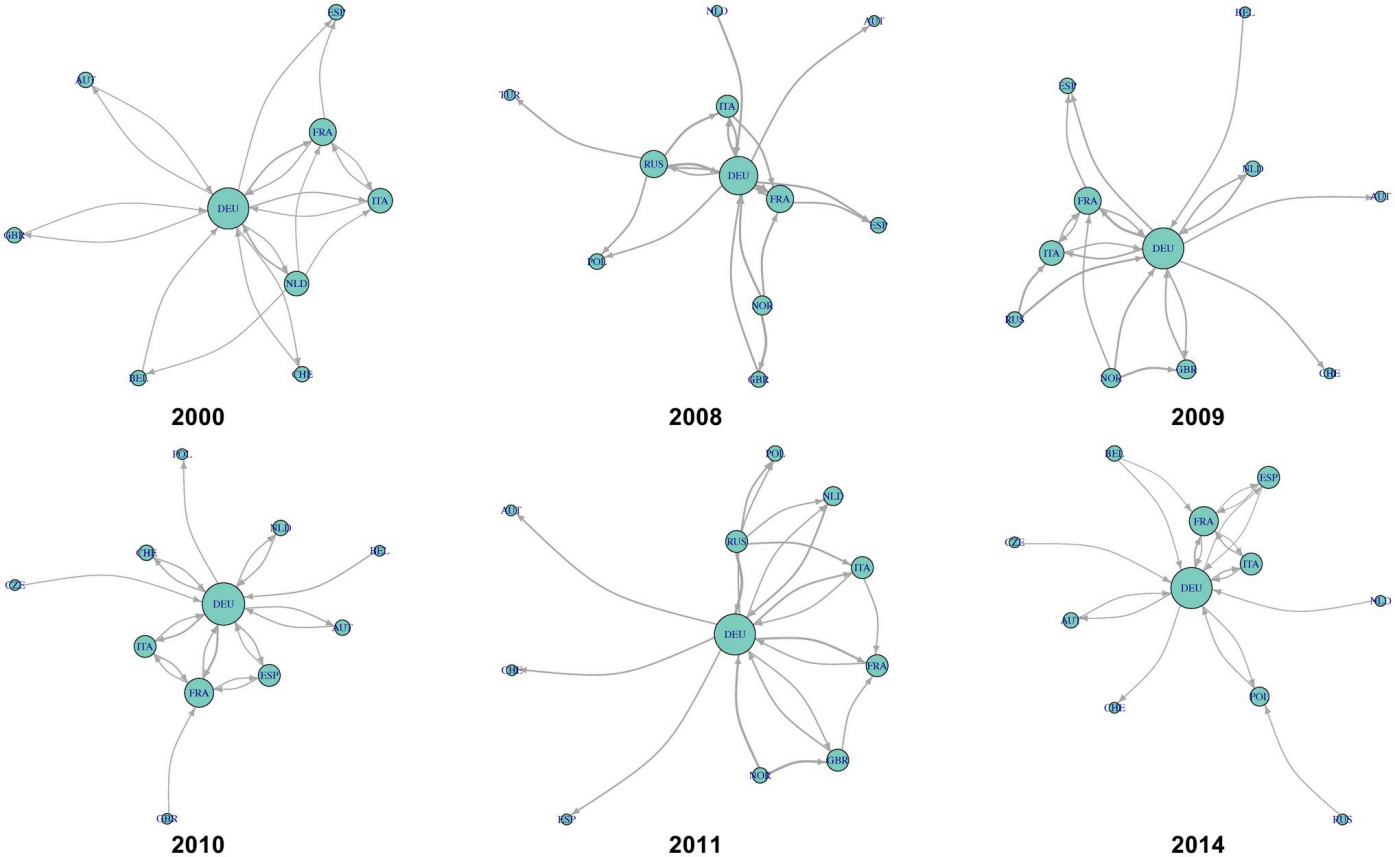
International value-added circulation of European community in 2000, 2008–2011, and 2014. These graphs illustrate the top 20 links. Their node sizes and link widths are in proportion to the square root of the degree and the amount of circular flow, respectively. S1 Table shows a detailed list of the sectors.

**Fig 13:**
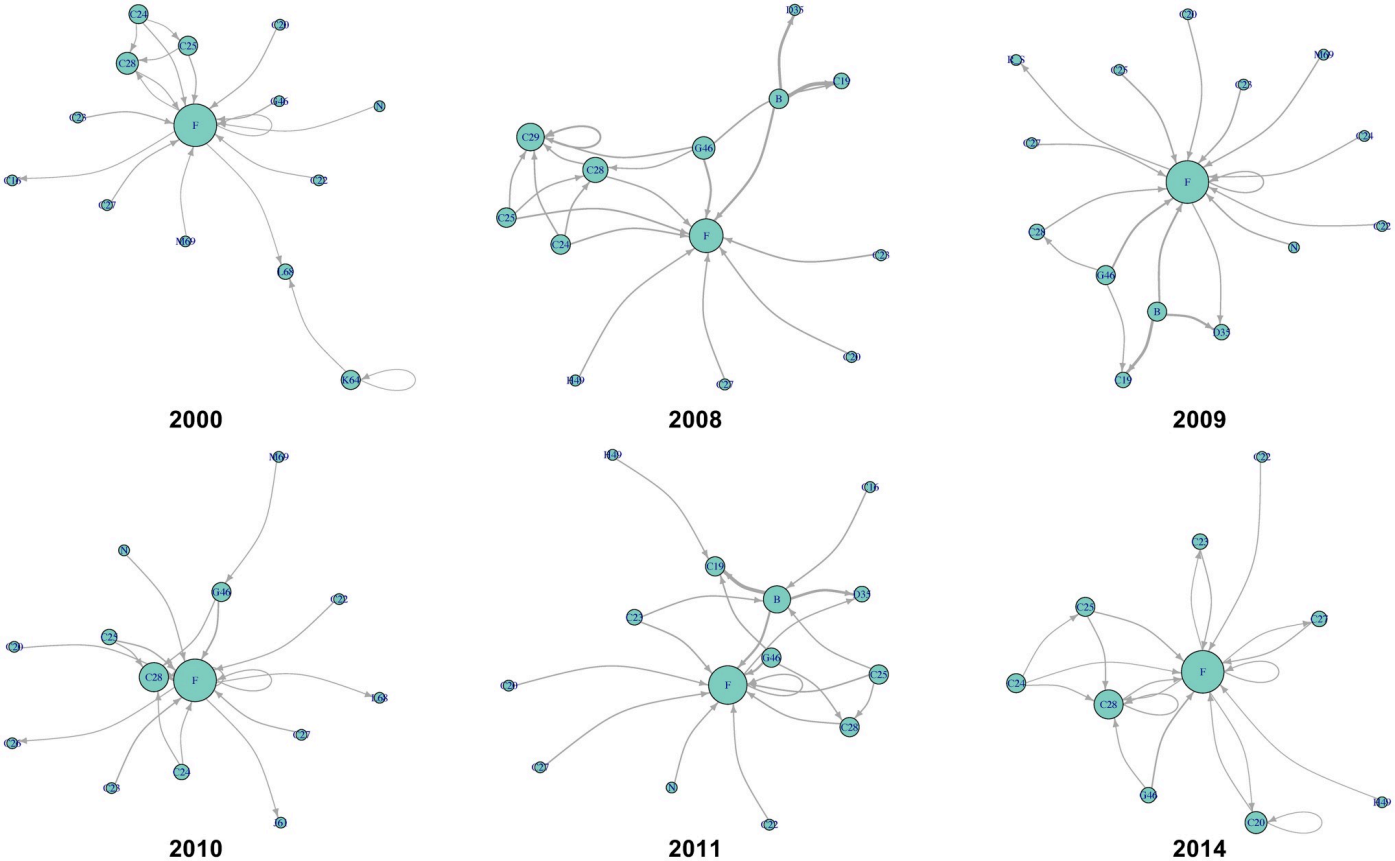
Intersectoral value-added circulation of European community in 2000, 2008–2011, and 2014. These graphs illustrate the top 20 links. Their node sizes and link widths are in proportion to the square root of the degree and the amount of circular flow, respectively. S2 Table shows a detailed list of the sectors.

To examine the implications of the economic integration index, we focused on the period of 2008–2011, which has exhibited substantial changes in economic integration (Fig 8); we also analyzed 2000 and 2014, which are the first and last years of the analyzed WIOD. Fig 12 shows the changes in the international and intersectoral relationships of circular flow in Europe. The circulation in 2008, the year of the highest integration index in Europe for the 15 years, is higher between Germany, France, Italy, and Russia compared with that in 2000. However, in 2011 and 2014, which has the lowest economic integration, Russia is absent in Europe, as shown in Tables 3 and 7. From a sectoral point of view, Fig 13 shows sector B: mining and quarrying as a hub of sectors F: construction; C19: manufacture of coke and refined petroleum products; and D35: electricity, gas, steam, and air conditioning supply. Therefore, the role of Russia and sector B: mining and quarrying is important for the high economic integration index in Europe.

**Fig 14:**
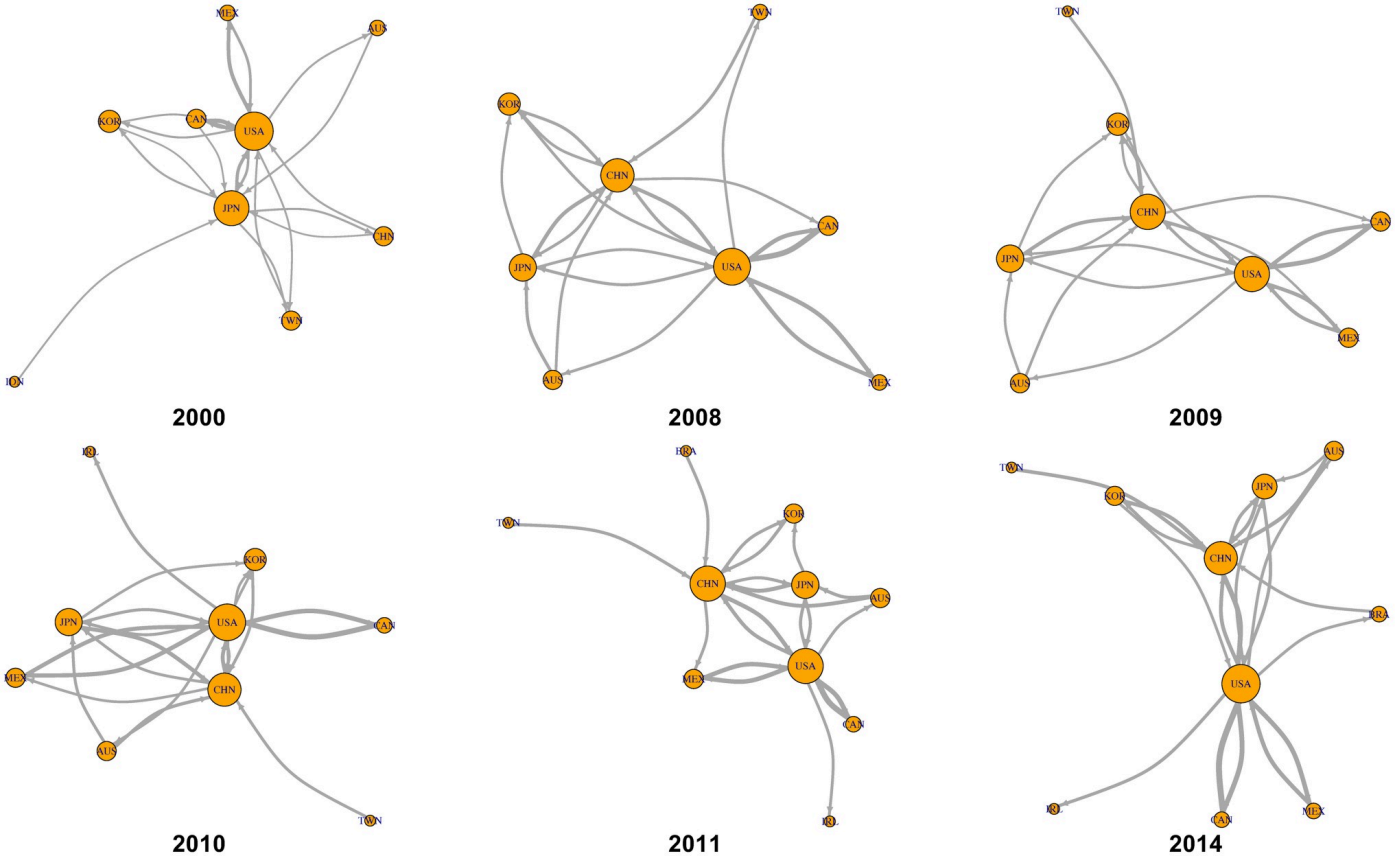
International value-added circulation of the Pacific Rim community in 2000, 2008–2011, and 2014. These graphs illustrate the top 20 links. Their node sizes and link widths are in proportion to the square root of the degree and the amount of circular flow, respectively. S1 Table shows a detailed list of the sectors.

**Fig 15:**
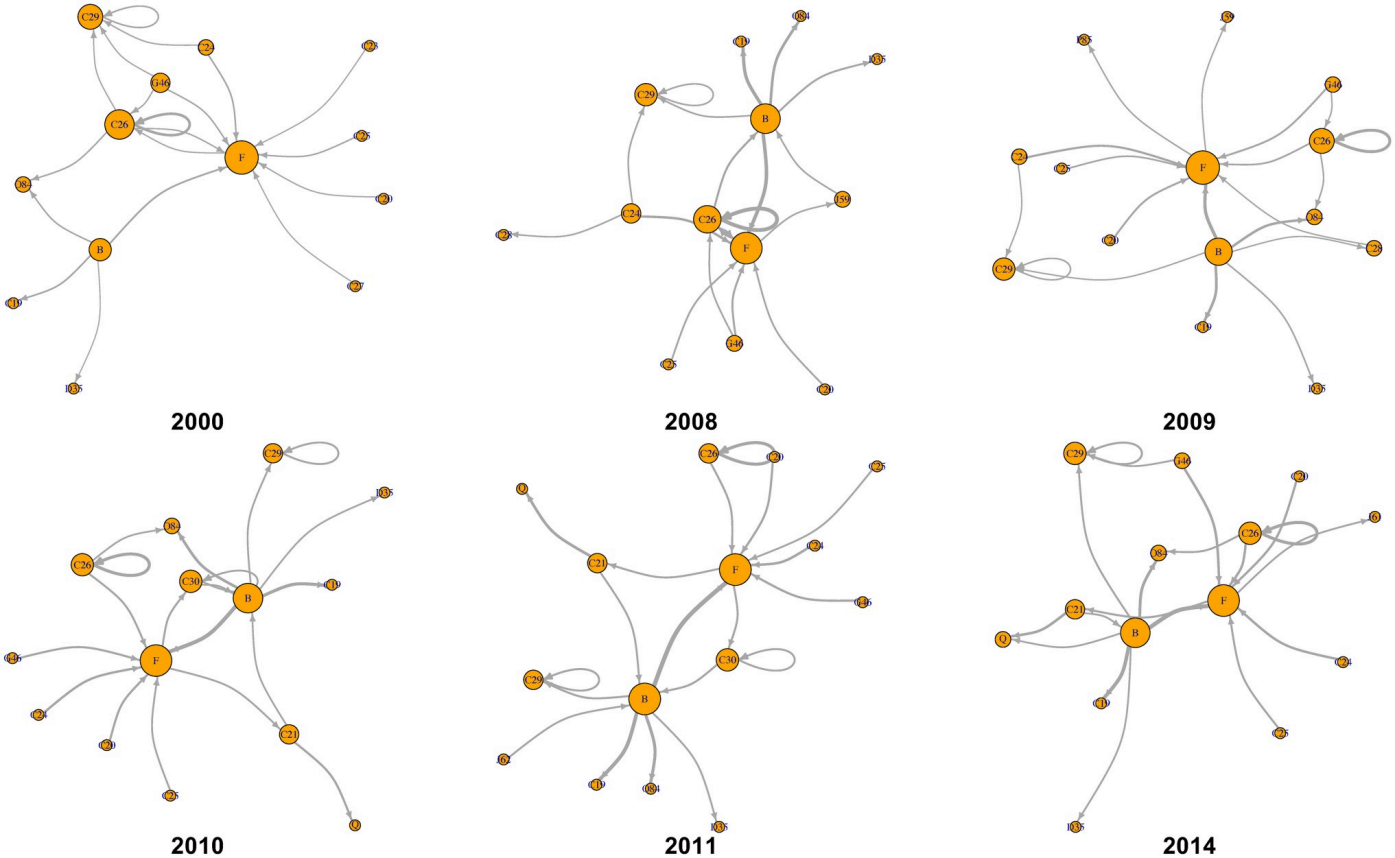
Intersectoral value-added circulation of the Pacific Rim community in 2000, 2008–2011, and 2014. These graphs illustrate the top 20 links. Their node sizes and link widths are in proportion to the square root of the degree and the amount of circular flow, respectively. S2 Table shows a detailed list of the sectors.

Figs 14 and 15 show the changes in international and intersectoral relationships in the Pacific Rim. According to Fig 14, the center of the value-added circulation in the Pacific Rim changed from the United States and Japan to the United States and China. This also means that the Pacific Rim, as a regional community of IVANs, was detected stably because of the strong value-added circulation around the United States and China despite the economic crisis around 2009. From the sectoral perspective, Fig 15 shows that there are three crucial points in the circular structure of the Pacific Rim; these are the strongest stable circulations from B: mining and quarrying to F: construction, those within C26: manufacture of computer, electronic, and optical products, and those within C29: manufacture of motor vehicles, and trailers and semi-trailers for the years 2000–2014.

In Europe, the circulation between sectors B: mining and quarrying and F: construction is unstable because sector B mainly occurs in Russia, which is not an EU member country. Therefore, the extent of economic integration tends to be unstable. By contrast, the Pacific Rim has mineral resources. It also has neutral- and low-potential sectors with high circulation, namely, manufacturing high-tech and motor vehicles, respectively, which produces the high-value-added products as parts of GVCs.

These results suggest that the stable growth of economic integration in the Pacific Rim has been driven by the relationship of the value-added circulation of these resources and international division in the manufacture of high value-added products. Furthermore, in the Pacific Rim, the international division of labor is advancing, and value-added circulation occurs across countries. This may be explained by the fact that, the free mobility of labor in the EU has led to the specialization of sectors; in the Pacific Rim, labor mobility is limited, and the international division of labor has led to the free movement of goods. The relationship between labor mobility and industrial structure is left for future research.

## Conclusion

The purpose of this study is to clarify how international economic integration is occurring from the perspective of trade in value-added. For this purpose, we used the WIOD released in 2016 to construct and analyze IVANs, which show the international relationship of sector-wise trade in value-added. First, the scope of economic integration was identified by Infomap, a community detection method that uses network flows. With threshold setting in the IVANs, regional communities in Europe and the Pacific Rim were detected throughout the 15 studied years. These two communities have not been found in studies that analyzed international trade networks constructed from the WIOD. To analyze how value flows within these two regions, we used Helmholtz–Hodge decomposition to extract the potential and circular relationships and clarified the annual changes in the roles played by the countries and sectors within these regions. In addition, we defined an economic integration index using the circular flow and applied it to both regions. We found that the level of economic integration in Europe, which had been increasing until 2008, dropped sharply after the economic crisis in 2009 to a level lower than that of the Pacific Rim in 2010, recovered in 2011, and dropped again after 2012 to a level below that of the Pacific Rim in 2013 and 2014. While the level of economic integration in Europe has been unstable, that in the Pacific Rim has been on a stable upward trend.

Moreover, the sectoral economic integration index provides a background to the changes in the extent of the economic integration of these two regions. In Europe, the extent of economic integration declined in 2009, 2010, and 2013 due to the decrease of intra-European value-added circulation through the industries of mining, construction, petroleum, metal, machinery manufacture, wholesale, financial services, and management services, which showed a large amount of the circular flow of IVAN. On the other hand, the economic integration index of the Pacific Rim was steady because the Pacific Rim community had stably included mining, manufacture (especially of motor vehicles and high-tech products), and construction which had high value-added circulation.

The obtained results suggest several topics for future research. The thresholds for detecting two regional communities by Infomap were significantly different before and after the economic crisis. What this means in terms of international trade requires further study. In addition, how to set appropriate thresholds for time-series community analysis of directed weighted networks such as IVANs also remains a research topic. Finally, it is also necessary to study whether the economic integration indices reflected the labor mobility in each regional community.

## Supporting information

S1 Table List of countries and regional classification. (PDF)

S2 Table List of sectors. (PDF)

S3 Table Number of communities of international trade networks. (PDF)

S1 Fig Community maps of international trade network. (PDF)
