# Supplementary material for: Regional economic integration via detection of circular flow in international value-added network
Source: PLoS One. 2021 Aug 20;16(8):e0255698. doi: 10.1371/journal.pone.0255698 (PMC8378758; doi:10.1371/journal.pone.0255698)
Supplement: S1 Table — (PDF) [file pone.0255698.s001.pdf]

S1 Table: List of countries and regional classification.

ISO code	Short name	Region	ISO code	Short name	Region
AUS	Australia	Pacific Rim	IRL	Ireland	Europe
AUT	Austria	Europe	ITA	Italy	Europe
BEL	Belgium	Europe	JPN	Japan	Pacific Rim
BGR	Bulgaria	Europe	KOR	Korea	Pacific Rim
BRA	Brazil	Pacific Rim	LTU	Lithuania	Europe
CAN	Canada	Pacific Rims	LUX	Luxembourg	Europe
CHE	Switzerland	Europe	LVA	Latvia	Europe
CHN	China	Pacific Rim	MEX	Mexico	Pacific Rim
CYP	Cyprus	Europe	MLT	Malta	Europe
CZE	Czechia	Europe	NLD	Netherlands	Europe
DEU	Germany	Europe	NOR	Norway	Europe
DNK	Denmark	Europe	POL	Poland	Europe
ESP	Spain	Europe	PRT	Portugal	Europe
EST	Estonia	Europe	ROU	Romania	Europe
FIN	Finland	Europe	RUS	Russia	Pacific Rim
FRA	France	Europe	SVK	Slovakia	Europe
GBR	United Kingdom	Europe	SVN	Slovenia	Europe
GRC	Greece	Europe	SWE	Sweden	Europe
HRV	Croatia	Europe	TUR	Turkey	Europe
HUN	Hungary	Europe	TWN	Taiwan	Pacific Rim
IDN	Indonesia	Pacific Rim	USA	America	Pacific Rim
IND	India	Pacific Rim			
