# Supplementary material for: Regional economic integration via detection of circular flow in international value-added network
Source: PLoS One. 2021 Aug 20;16(8):e0255698. doi: 10.1371/journal.pone.0255698 (PMC8378758; doi:10.1371/journal.pone.0255698)
Supplement: S2 Table — (PDF) [file pone.0255698.s002.pdf]

S2 Table: List of sectors.

Code	Description
A01	Crop and animal production, hunting and related service activities
A02	Forestry and logging
A03	Fishing and aquaculture
B	Mining and quarrying
C10	Manufacture of food products, beverages and tobacco products
C13	Manufacture of textiles, wearing apparel and leather products
C16	Manufacture of wood and of products of wood and cork except furniture, and manufacture of articles of straw and plaiting materials
C17	Manufacture of paper and paper products
C18	Printing and reproduction of recorded media
C19	Manufacture of coke and refined petroleum products
C20	Manufacture of chemicals and chemical products
C21	Manufacture of basic pharmaceutical products and pharmaceutical preparations
C22	Manufacture of rubber and plastic products
C23	Manufacture of other non-metallic mineral products
C24	Manufacture of basic metals
C25	Manufacture of fabricated metal products except machinery and equipment
C26	Manufacture of computer, electronic, and optical products
C27	Manufacture of electrical equipment
C28	Manufacture of machinery and equipment n.e.c.
C29	Manufacture of motor vehicles, trailers and semi-trailers
C30	Manufacture of other transport equipment
C31	Manufacture of furniture, and other manufacturing
C33	Repair and installation of machinery and equipment
D35	Electricity, gas, steam and air conditioning supply
E36	Water collection, treatment and supply
E37	Sewerage, waste collection, treatment and disposal activities, and materials recovery, and remediation activities and other waste management services
F	Construction
G45	Wholesale and retail trade and repair of motor vehicles and motorcycles
G46	Wholesale trade except that of motor vehicles and motorcycles
G47	Retail trade except that of motor vehicles and motorcycles
H49	Land transport and transport via pipelines
H50	Water transport
H51	Air transport
H52	Warehousing and support activities for transportation
H53	Postal and courier activities
I	Accommodation and food service activities
J58	Publishing activities
J59	Motion picture, video and television programme production, sound recording and music publishing activities; programming and broadcasting activities
J61	Telecommunications
J62	Computer programming, consultancy and related activities; information service activities
K64	Financial service activities, except insurance and pension funding
K65	Insurance, reinsurance and pension funding except compulsory social security
K66	Activities auxiliary to financial services and insurance activities
L68	Real estate activities
M69	Legal and accounting activities, and activities of head offices, and management consultancy activities
M71	Architectural and engineering activities, and technical testing and analysis
M72	Scientific research and development
M73	Advertising and market research
M74	Other professional, scientific and technical activities, and veterinary activities
N	Administrative and support service activities
O84	Public administration and defense, and compulsory social security
P85	Education
Q	Human health and social work activities
R_S	Other service activities
T	Activities of households as employers, and undifferentiated goods and services producing activities of households for own use
U	Activities of extraterritorial organizations and bodies
