# Supplementary material for: Regional economic integration via detection of circular flow in international value-added network
Source: PLoS One. 2021 Aug 20;16(8):e0255698. doi: 10.1371/journal.pone.0255698 (PMC8378758; doi:10.1371/journal.pone.0255698)
Supplement: S3 Table — (PDF) [file pone.0255698.s003.pdf]

S3 Table: Number of communities of international trade networks*.

Year	Number of communities	Sizes of the communities.
2000	10	1713, 123, 94, 93, 52, 52, 46, 43, 26, 18
2001	8	1881, 116, 58, 55, 44, 40, 37, 23
2002	9	1657, 153, 120, 101, 60, 55, 44, 44, 23
2003	13	1494, 114, 110, 100, 87, 66, 53, 52, 51, 47, 39, 22, 14
2004	15	1509, 111, 90, 89, 67, 59 56, 50, 47, 44, 38, 24, 23, 20, 14
2005	9	1891, 110, 51, 50, 43, 40, 35, 22, 12
2006	10	1798, 119, 64, 53, 47, 45, 44, 38, 29, 14
2007	13	1482, 144, 116, 93, 71, 66, 52, 52, 51, 44, 34, 29, 14
2008	14	1350, 165, 146, 105, 93, 86, 70, 52, 44, 43, 36, 35, 21, 12
2009	9	1880, 113, 67, 57, 43, 37, 24, 15, 14
2010	10	1712, 111, 109, 71, 64, 55, 43, 36, 36, 10
2011	13	979, 561, 111, 109, 101, 70, 65, 59, 47, 44, 37, 33, 13
2012	14	1421, 116, 112, 101, 91, 64, 63, 58, 55, 44, 43, 31, 22, 21
2013	12	1581, 179, 155, 68, 55, 52, 47, 45, 26, 16, 11, 10
2014	15	1238, 220, 158, 122, 116, 65, 55, 55, 46, 41, 36, 33, 28, 24, 10

* This table shows the results of the Infomap analysis [32, 36] of the international trade network excluding domestic transactions as well as IVAN. The hierarchy of the results of Infomap was two levels and these communities are the first layer. The data is from WIOD [5, 6, 7]. IVAN detected only one huge community, while ITN detected many communities even without setting any threshold. In the table, only communities with more than 10 nodes are counted, and the number of nodes for each is shown in order of increasing size. Looking at the size, one community is relatively large every year. The smallest maximum community size was in 2011, and the largest is in 2005. The maximum communities include service sectors and the second and subsequent communities are sectors of manufactures or food industries. In other words, the same type of industry has been detected as the ITN community. Detailed results of the community analysis are summarized in S1 Figure.
