# Supplementary material for: Regional economic integration via detection of circular flow in international value-added network
Source: PLoS One. 2021 Aug 20;16(8):e0255698. doi: 10.1371/journal.pone.0255698 (PMC8378758; doi:10.1371/journal.pone.0255698)

S1 Figure: Community maps of international trade network*.

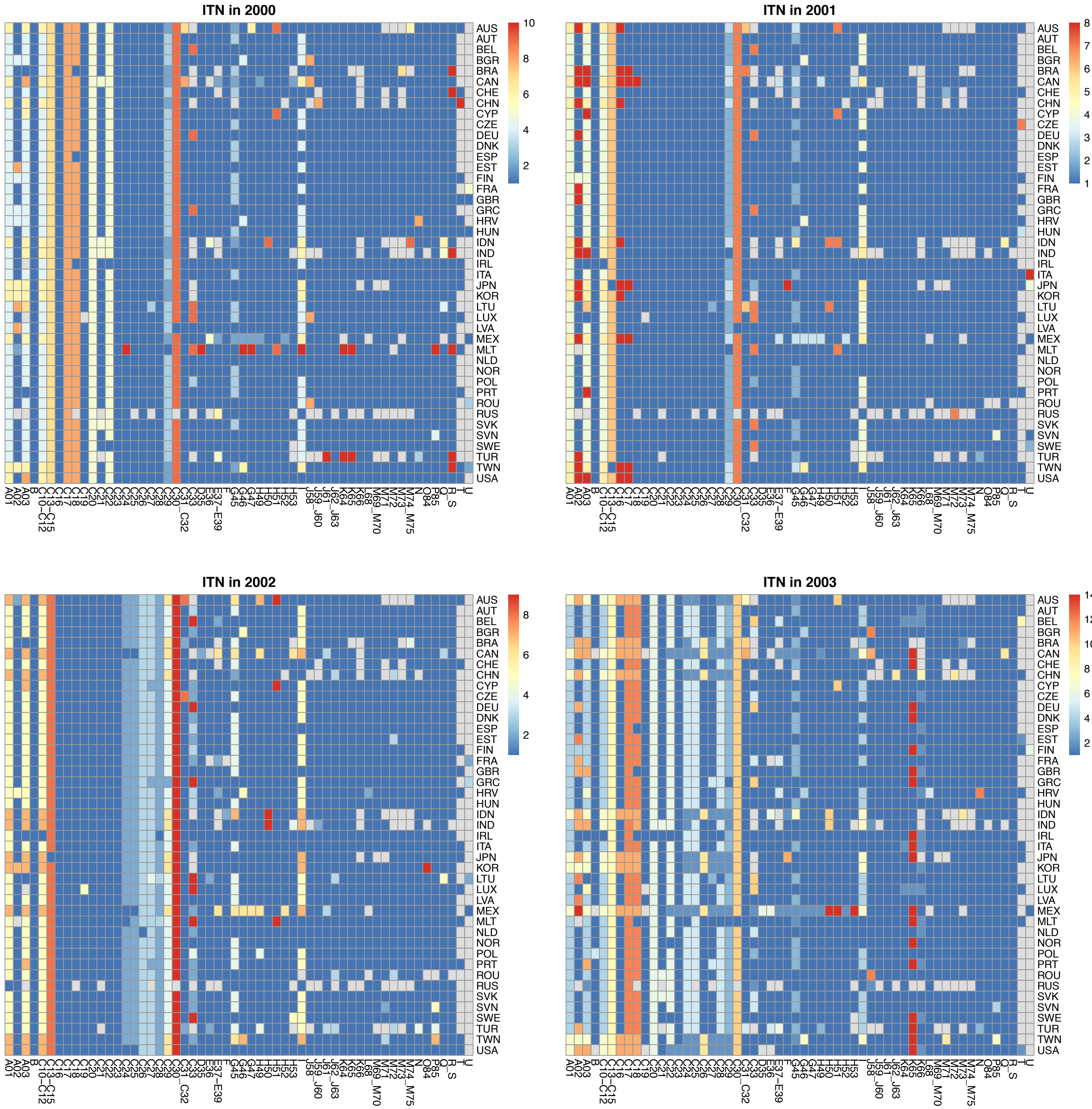

* Each cell means node of ITN and the row and column of the heat maps are countries and sectors. The legend shows ID of communities, which means colors represent communities' ID. We obtained IDs to communities whose size is more than 10 nodes and the gray cells are nodes belonging to communities which are smaller than 10.

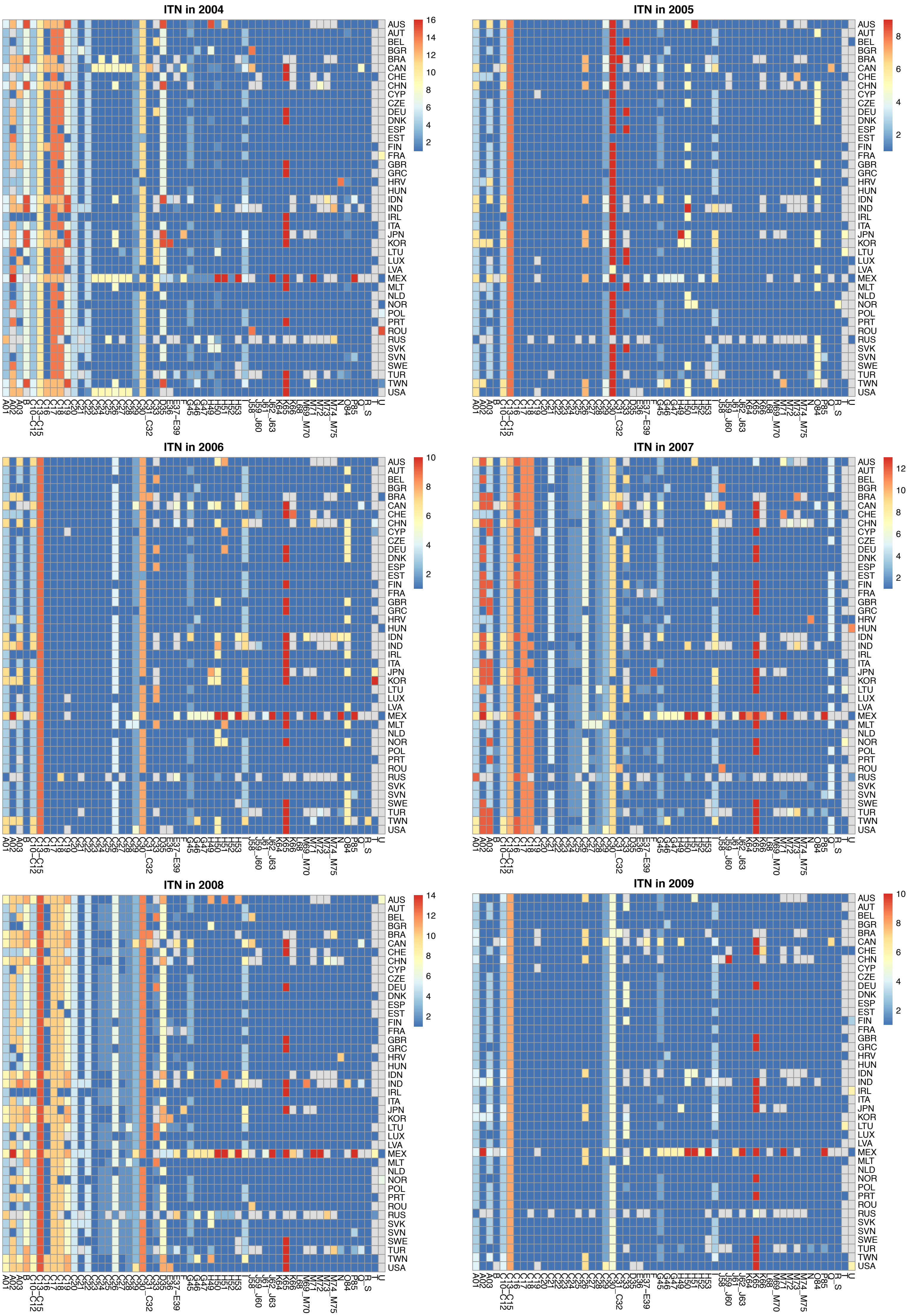
ITN in 2004
ITN in 2005
ITN in 2006
ITN in 2007
ITN in 2008
ITN in 2009
AUS AUT BEL BGR BRA CAN CHE CHN CYP CZE DEU DNK ESP EST FIN FRA GBR GRC HRV HUN IDN IND IRL ITA JPN KOR LTU LUX LVA MEX MLT NLD NOR POL PRT ROU RUS SVK SVN SWE TUR TWN USA
A01 A02 A03 B C10–C12 C13–C15 C16 C17 C18 C19 C20 C21 C22 C23 C24 C25 C26 C27 C28 C29 C30 C31_C32 C33 D35 E36 E37–E39 F G45 G46 G47 H49 H50 H51 H52 H53 I J58 J59_J60 J61 J62_J63 K64 K65 K66 L68 M69_M70 M71 M72 M73 M74_M75 N O84 P85 Q R_S T U

ITN in 2010

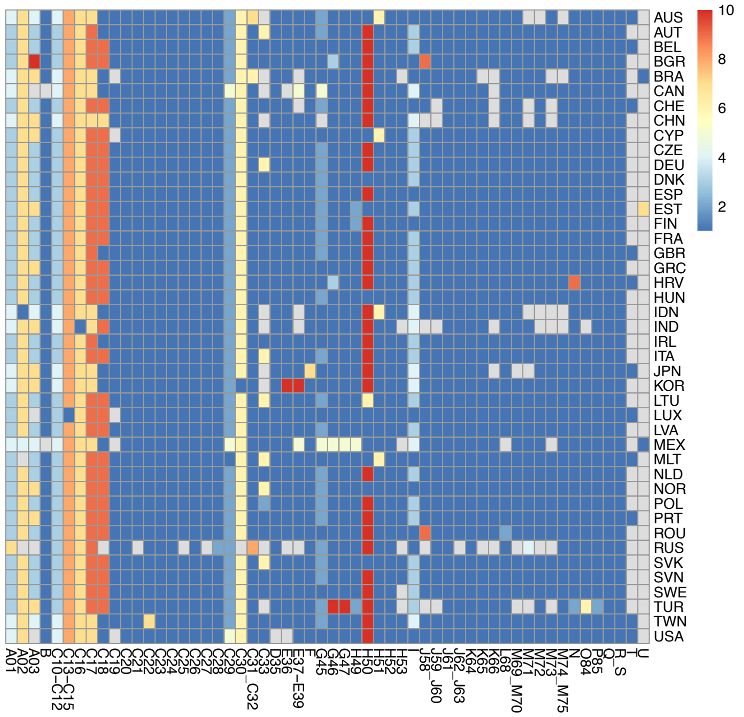

ITN in 2011

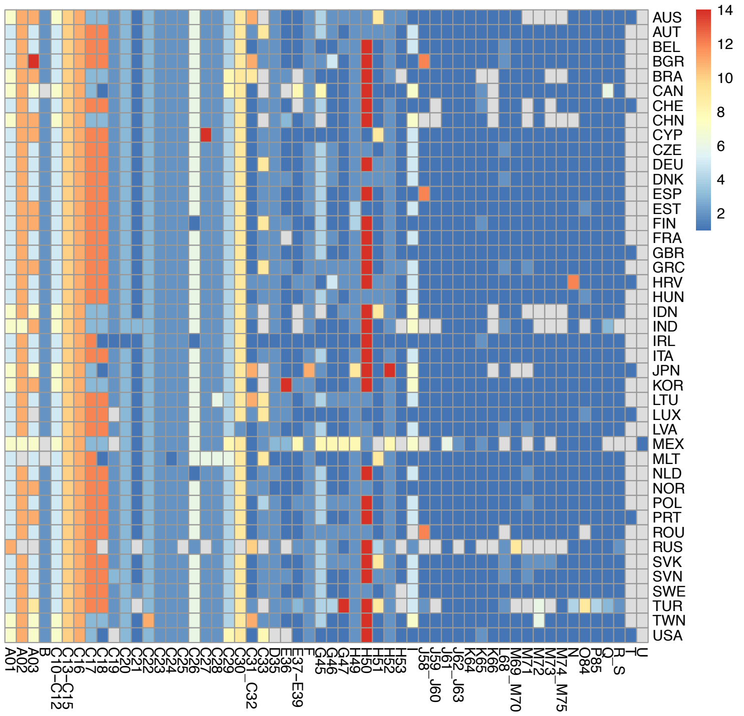

ITN in 2012

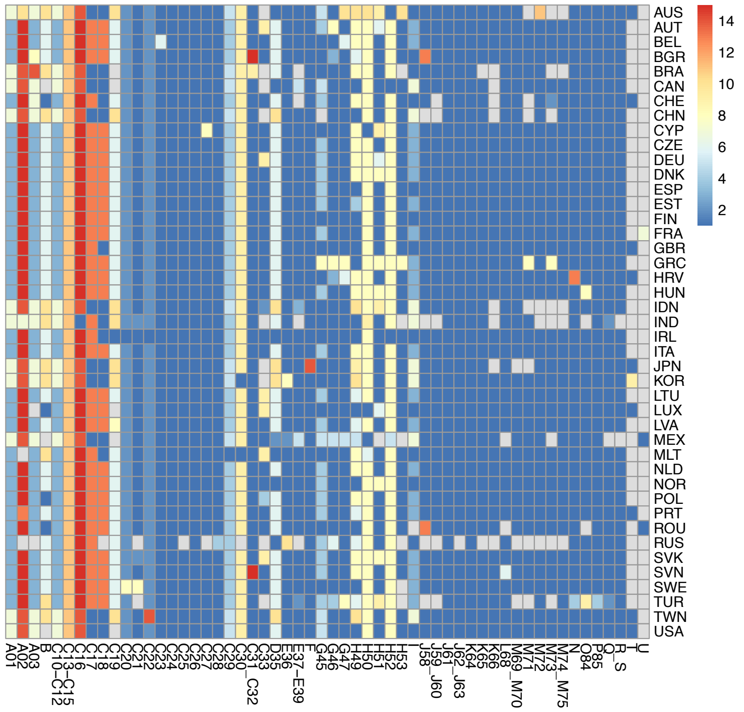

ITN in 2013

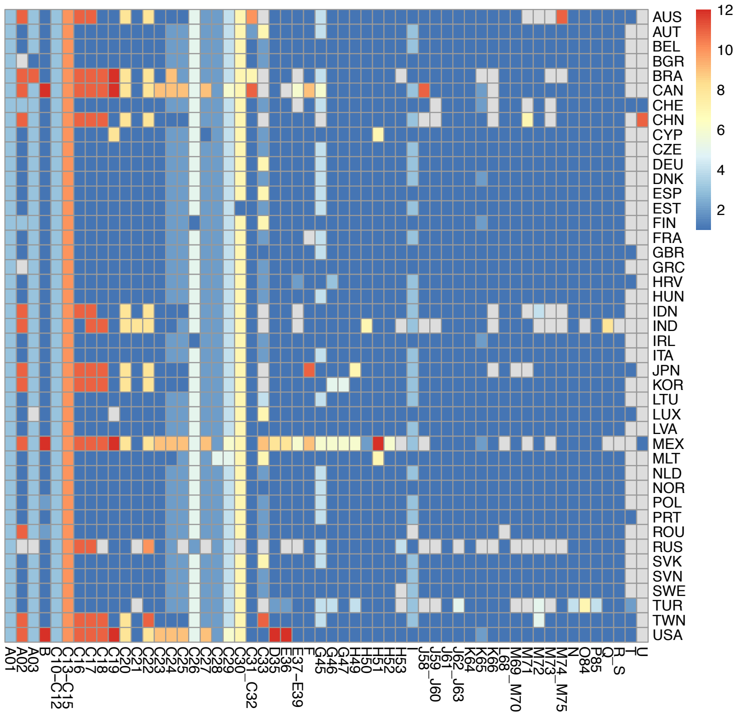

ITN in 2014

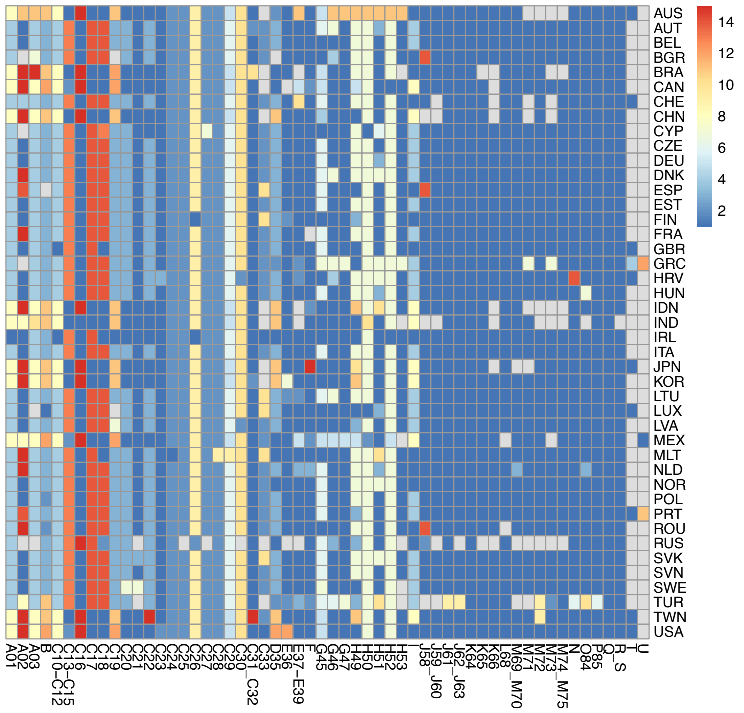

Supplement: S1 Fig — (PDF) [file pone.0255698.s004.pdf]
